# Varicella zoster virus glycoprotein E facilitates PINK1/Parkin-mediated mitophagy to evade STING and MAVS-mediated antiviral innate immunity

**DOI:** 10.1038/s41419-023-06400-z

**Published:** 2024-01-06

**Authors:** Soo-Jin Oh, Je-Wook Yu, Jin-Hyun Ahn, Seok Tae Choi, Hosun Park, Jeanho Yun, Ok Sarah Shin

**Affiliations:** 1 BK21 Graduate Program, Department of Biomedical Sciences, College of Medicine, Korea University Guro Hospital, Seoul, Republic of Korea; 2 Department of Microbiology and Immunology, Institute for Immunology and Immunological Diseases, Yonsei University College of Medicine, Seoul, Republic of Korea; 3 Department of Microbiology, Sungkyunkwan University School of Medicine, Suwon, Republic of Korea; 4 Department of Microbiology, College of Medicine, Yeungnam University, Daegu, Republic of Korea; 5 Department of Translational Biomedical Sciences, Graduate School of Dong-A University, Busan, Republic of Korea

**Keywords:** Infection, Energy metabolism, Viral infection

## Abstract

Viruses have evolved to control mitochondrial quality and content to facilitate viral replication. Mitophagy is a selective autophagy, in which the damaged or unnecessary mitochondria are removed, and thus considered an essential mechanism for mitochondrial quality control. Although mitophagy manipulation by several RNA viruses has recently been reported, the effect of mitophagy regulation by varicella zoster virus (VZV) remains to be fully determined. In this study, we showed that dynamin-related protein-1 (DRP1)-mediated mitochondrial fission and subsequent PINK1/Parkin-dependent mitophagy were triggered during VZV infection, facilitating VZV replication. In addition, VZV glycoprotein E (gE) promoted PINK1/Parkin-mediated mitophagy by interacting with LC3 and upregulating mitochondrial reactive oxygen species. Importantly, VZV gE inhibited MAVS oligomerization and STING translocation to disrupt MAVS- and STING-mediated interferon (IFN) responses, and PINK1/Parkin-mediated mitophagy was required for VZV gE-mediated inhibition of IFN production. Similarly, carbonyl cyanide m-chlorophenyl hydrazone (CCCP)-mediated mitophagy induction led to increased VZV replication but attenuated IFN production in a three-dimensional human skin organ culture model. Our results provide new insights into the immune evasion mechanism of VZV gE via PINK1/Parkin-dependent mitophagy.

## Introduction

Mitochondria are highly dynamic organelles that participate in multiple biological processes including ATP production, metabolism, cell death, and inflammation. In addition, mitochondria serve as antiviral immune platforms [1–3]. Mitochondrial quality control is responsible for maintaining function and homeostasis, and operates by coordinating various processes ranging from mitochondrial dynamics to mitophagy. In particular, damaged or dysfunctional mitochondria can be selectively degraded via mitophagy, which forms mitochondria-containing autophagosomes by fusing with lysosomes [4]. Multiple viruses subvert cellular antiviral activities by manipulating mitophagy for persistence and replication at various stages [5]. However, studies involving molecular mechanisms and functional implications of mitophagy during viral infections have been limited.

Mitophagy can be initiated by the loss of mitochondrial membrane potential (MMP). Subsequently, PTEN-induced kinase 1 (PINK1) is stabilized on the outer mitochondrial membrane, where it phosphorylates an E3 ubiquitin ligase, Parkin. Consequently, activated Parkin ubiquitinates substrates such as mitofusin 1/2 (MFN1/2), Miro, and VDAC, which function as autophagy-mediated degradation signals, on damaged mitochondria. Recent studies have suggested that PINK1/ubiquitin-independent pathway can be activated by mitophagy receptors, such as BNIP3, BNIP3L/NIX, FUNDC1, and optineurin (OPTN) under certain physiological conditions [4, 6]. Given the close association between defective mitophagy and a wide range of diseases, including neurodegenerative diseases and cancer, it is important to clarify the role of mitophagy during viral infection.

Varicella zoster virus (VZV) is a medically important herpesvirus, that causes chickenpox during primary infection and exhibits T cell and skin tropism [7]. Interestingly, VZV can establish a latent infection in the dorsal root ganglia, and VZV reactivation can cause zoster accompanied by pain. Despite the introduction of the live-attenuated chickenpox vaccine in the early 1990s and the recent introduction of the subunit zoster vaccine, VZV remains highly prevalent worldwide. Although previous studies have shown the activation of autophagy in VZV-infected dermal cells and tissues [8–10], the potential role of skin-mediated mitochondrial dynamics and mitophagy during VZV infection has not yet been described.

VZV glycoprotein E (gE), encoded by open reading frame (ORF) 68 (ORF68), is the most abundant glycoprotein expressed on the surface of VZV and thus acts as a major antigen and vaccine immunogen [11]. The multifaceted role of gE has been previously studied. As an example, VZV gE binds to insulin-degrading enzymes to promote VZV infectivity [12] and is critical for T cell tropism and neurovirulence [13, 14]. Recently, the recombinant subunit vaccine Shingrix™, based on gE protein and AS01 adjuvant, was found to be superior to the attenuated vaccine Zostavax® in terms of both safety and efficacy [15]. Although VZV gE is an important virulence factor, its effect on mitochondrial dynamics is poorly understood.

In this study, we examined whether VZV infection disrupts mitochondrial dynamics and induces mitophagy in human skin cells and skin organ culture (SOC) model. Furthermore, we found that VZV gE is a key regulator of PINK1/Parkin-dependent mitophagy by interacting with LC3, triggering mitochondrial reactive oxygen species (ROS) (mtROS) production, and abolishing STING- and mitochondrial antiviral signaling protein (MAVS)-mediated IFN responses. Our findings further highlight the importance of mitophagy in controlling VZV infection and the role of VZV gE as an IFN antagonist.

## Results

### VZV infection activates PINK1/Parkin-dependent mitophagy

**Fig. 1:**
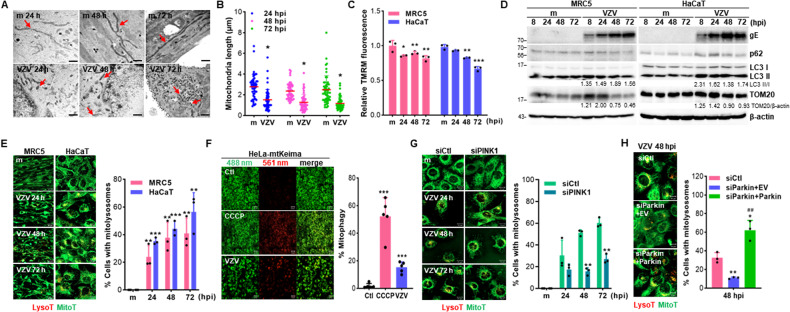
VZV infection results in mitochondrial fission and activates PINK1/Parkin-dependent mitophagy. **A** MRC5 cells were infected with mock (m) or VZV YC01 (MOI 0.001). Representative transmission electron microscope images of mock or VZV-infected cells. The red arrow indicates mitochondria. Scale bar = 1 μm. **B** Quantitative analysis of mitochondria lengths in mock (m) vs. VZV-infected MRC5 cells. The length of ten mitochondria per cell present in more than five cells in each group was measured. Statistical analysis, *p < 0.05 vs. mock-infected cells. **C** MRC5 and HaCaT cells were infected with mock (m) or VZV (MOI 0.001) for indicated times. Relative tetramethylrhodamine methyl ester (TMRM) intensity was measured (mean ± SD; *n* = 3). *p < 0.05; **p < 0.01; ***p < 0.001 vs. mock-infected cells. **D** Immunoblot analysis of VZV gE, p62, LC3, and TOM20 expression in VZV (MOI 0.001)-infected cells at the indicated time points. β-actin was used as a protein loading control. Numbers below the blot represent quantified band intensity by densitometric analysis. **E** MRC5 and HaCaT cells were infected with mock (m) or VZV (MOI 0.001) and subsequently stained with MitoTracker (green) and LysoTracker (red). % cells with LysoTracker and MitoTracker colocalization were calculated and are presented in the graph. **p < 0.01; ***p < 0.001 vs. mock-infected cells. **F** Representative confocal analysis of VZV-infected HeLa-Parkin cells expressing mtKeima. Cells were treated with 25 μM CCCP for 2 h or infected with VZV for 48 h. Representative mtKeima fluorescence images are shown and the graph on the right demonstrates mitophagy index. ***p < 0.001 vs. mock-infected control cells (Ctl). **G** Representative confocal images of HaCaT cells transfected with siCtl or siPINK1, followed by VZV infection (MOI 0.001). The graph shows the percentage of cells displaying mitolysosomes by assessing LysoTracker and MitoTracker colocalization. A minimum of one hundred cells per condition were counted in three independent experiments. **p < 0.01 vs. siCtl-expressing mock-infected cells. **H** HaCaT cells were transfected with control (siCtl) or Parkin siRNA (siParkin), followed by empty vector (EV) or Parkin-MYC plasmids (siParkin+Parkin) transfection. After transfection, cells were infected with VZV (MOI 0.001) and stained with LysoTracker and MitoTracker to examine the formation of mitolysosomes at 48 hpi. *p < 0.05; **p < 0.01 vs. siCtl-expressing cells, ##*p* < 0.01 vs. siParkin-expressing EV-transfected cells.

The role of mitophagy during VZV infection has not been investigated. Thus, we first examined mitochondrial morphology upon VZV infection using transmission electron microscopy. Interestingly, at 24 h post-infection (hpi), there were double-membrane vesicles partially containing mitochondria and at 48 hpi, disrupted mitochondria-containing autophagosome significantly increased upon VZV infection (Fig. S1). Simultaneously, we found the length of mitochondria per cell significantly decreased in response to VZV (Fig. 1A, B). Additionally, a significant loss of MMP was detected in both VZV-infected MRC5 and HaCaT cells (Fig. 1C). Next, we observed that VZV infection led to activation of autophagy-related proteins in a time-dependent manner, as shown by increased LC3 II-I ratio and decreased p62 expression (Fig. 1D), whereas VZV infection suppressed the expression of the mitochondrial outer membrane protein TOM20.

To explore potential mechanisms of VZV-mediated mitophagy induction, mitolysosome formation following VZV infection was examined by confocal microscopy. Both MRC5 and HaCaT cells were infected with VZV, followed by staining with LysoTracker Red and MitoTracker Green, which are fluorescent probes widely used for staining lysosomes and mitochondria, respectively. The percentage of cells containing mitolysosomes increased in a time-dependent manner in response to VZV infection (Fig. 1E). Additionally, we quantitatively measured mitophagy using mtKeima, a pH-sensitive dual-excitation ratio-metric fluorescent protein that exhibits resistance to lysosomal proteases [16]. VZV infection in HeLa cells stably expressing parkin labeled mtKeima (HeLa-mtKeima) cells led to a significant augmentation in mitophagy levels, comparable to that of Carbonyl cyanide m-chlorophenyl hydrazone (CCCP) treatment (Fig. 1F). CCCP was used as a positive control for mitophagy induction. CCCP treatment of MRC5 and HaCaT cells did not result in toxicity but increased the number of mitolysosomes formed (Fig. S2A, B). To determine whether VZV induction of mitophagy requires PINK1/Parkin, we introduced siRNAs specific to control, PINK1 or Parkin in HaCaT cells following VZV infection. PINK1 expression was significantly inhibited upon PINK1-specific siRNA transfection with or without VZV infection (Fig. S3A, B). Moreover, Parkin expression was diminished in siRNA-transfected cells, but rescued by Parkin-encoding plasmid transfection (Fig. S3C). Mitolysosome formation was greatly attenuated by PINK1 or Parkin knockdown compared to that in control siRNA-transfected cells, highlighting the importance of PINK1/Parkin in mitophagy induction by VZV (Fig. 1G, H).

### PINK1-mediated mitophagy facilitates VZV replication by attenuating IFNs

**Fig. 2:**
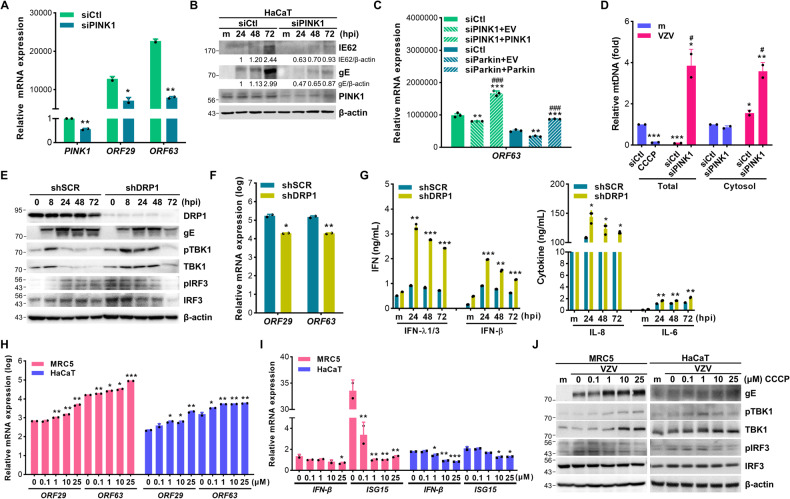
Mitochondrial fission and PINK1-mediated mitophagy facilitate VZV replication. **A** HaCaT cells expressing control (siCtl) or PINK1 siRNA (siPINK1) were infected with VZV (MOI 0.001) for 48 h. VZV ORF29 and ORF63 gene expression was examined and analyzed by RT-qPCR (mean ± SD; *n* = 3). *p < 0.05; **p < 0.01 vs. siCtl-expressing cells. **B** Knockdown efficiency of PINK1 was confirmed and VZV IE62 and gE expression were measured by immunoblot analysis. **C** HaCaT cells were transfected with PINK1-(siPINK1) or Parkin-specific siRNA (siParkin) followed by EV, PINK1-V5 (siPINK1 + PINK1), or Parkin-MYC (siParkin+Parkin) plasmids transfection. After infection with VZV (MOI 0.001) for 48 h, the gene expression level of VZV ORF63 was determined by RT-qPCR (mean ± SD; *n* = 3). **p < 0.01; ***p < 0.001 vs. siCtl-expressing cells. ###p < 0.001 vs. siPINK1 or siParkin-expressing cells. **D** To quantify the mitochondrial DNA (mtDNA) content, genomic DNA was extracted from cell lysate (Total) or cytosol (Cytosol) solution in 25 μM CCCP treated or VZV (MOI 0.001)-infected HaCaT cells for 48 h. Relative mtDNA level was analyzed by RT-qPCR (mean ± SD; *n* = 3). *p < 0.05; **p < 0.01; ***p < 0.001 vs. mock-infected DMSO-treated cells. #*p* < 0.05 vs. siCtl-expressing VZV-infected cells. **E** Scrambled control shRNA (shSCR) and DRP1-specific shRNA-expressing (shDRP1) THP-1 cells were infected with VZV (MOI 0.001) for the indicated times. Protein levels of DRP1, VZV gE, phospho-TBK1, TBK1, phospho-IRF3, and IRF3 were measured, and a representative blot was shown. **F** ShSCR and shDRP1 THP-1 cells were infected with VZV (MOI 0.001) for 48 h. VZV ORF29 and ORF63 gene expression levels were quantitated, normalized, and analyzed by RT-qPCR (mean ± SD; *n* = 3). *p < 0.05, **p < 0.01 vs. VZV-infected shSCR cells. **G** IFN-β, IFN-λ1/3, IL-8, and IL-6 secretion levels were measured by ELISA. *p < 0.05, **p < 0.01, ***p < 0.001 vs. mock- or VZV-infected shSCR cells at the indicated time points. **H**, **I** Cells were pre-treated with either DMSO or different doses of CCCP for 2 h and infected with VZV (MOI 0.001) for 48 h. Host and viral gene expression was quantitated, normalized, and analyzed by RT-qPCR (mean ± SD; *n* = 3). *p < 0.05; **p < 0.01; ***p < 0.001 vs. DMSO-treated cells. **J** Expression of VZV gE, phospho-TBK1, TBK1, phospho-IRF3, and IRF3 were measured by immunoblot analysis.

Next, we examined the role of PINK1 during VZV replication by measuring VZV ORF29 (early gene) and ORF63 (immediate early gene) expression in PINK1-knockdown or rescued cells. PINK1 expression was checked by immunoblot assays which showed decreased PINK1 expression level in PINK1-knockdown cells and increased PINK1 expression in PINK1-rescued cells (Fig. S3D). There was a significant reduction of VZV IE62 and gE protein levels as well as ORF29 and ORF63 gene expression in PINK1-knockdown cells (Fig. 2A, B). Similarly, Parkin deficiency significantly reduced ORF63 expression but it was rescued by Parkin overexpression (Fig. 2C). Furthermore, VZV infection diminished mtDNA volume, similar to that in the case of CCCP-induced mitophagy; however, mitochondrial DNA (mtDNA) volume recovered by the loss of PINK1 (Fig. 2D). In terms of cytosolic mtDNA, VZV infection slightly increased cytosolic mtDNA leakage and PINK1 knockdown cells showed significantly increased mtDNA leakage to the cytosol, suggesting that PINK1 prevented mtDNA leakage into the cytosol in response to VZV.

Dynamin-related protein-1 (DRP1)-dependent mitochondrial fission is initiated by mitochondrial membrane depolarization. To determine the role of DRP1-dependent mitochondrial fission during VZV infection, scrambled control shRNA (shSCR) and DRP1-specific shRNA (shDRP1) THP-1 cells were prepared and infected with VZV. The lack of DRP1 led to the attenuation of VZV gE protein expression as well as ORF29 and ORF63 gene expression while upregulating the phosphorylation of TANK-binding kinase 1 (TBK1) and interferon regulatory factor 3 (IRF3) (Fig. 2E, F). Along with the activation of TBK1 and IRF3, DRP1 deficiency upregulated both type I and III IFN secretion as well as the levels of pro-inflammatory cytokines, such as IL-6 and IL-8, in response to VZV (Fig. 2G).

To characterize whether CCCP, which is known to trigger mitochondrial depolarization, affects viral replication, MRC5 and HaCaT cells were pre-incubated with different doses of CCCP and then infected with VZV for 48 h. CCCP treatment significantly augmented VZV gene expression in a dose-dependent manner (Fig. 2H). Interestingly, CCCP elicited an opposite effect on host genes such as IFN-β and ISG15, both of which are involved in host defense against viral infection, suggesting that CCCP-mediated mitochondrial depolarization regulates IFN pathway as well as viral replication (Fig. 2I). Next, we examined the cGAS/STING pathway, which is essential for the induction of IFN. VZV-induced phosphorylation of TBK1 and IRF3 was significantly downregulated in a CCCP dose-dependent manner (Fig. 2J). Taken together, these results indicate that PINK1-mediated mitophagy enhanced viral replication by inhibiting the mtDNA-induced antiviral pathway during VZV infection.

### VZV gE modulates mitochondria dynamics and regulates PINK1/Parkin-dependent mitophagy

**Fig. 3:**
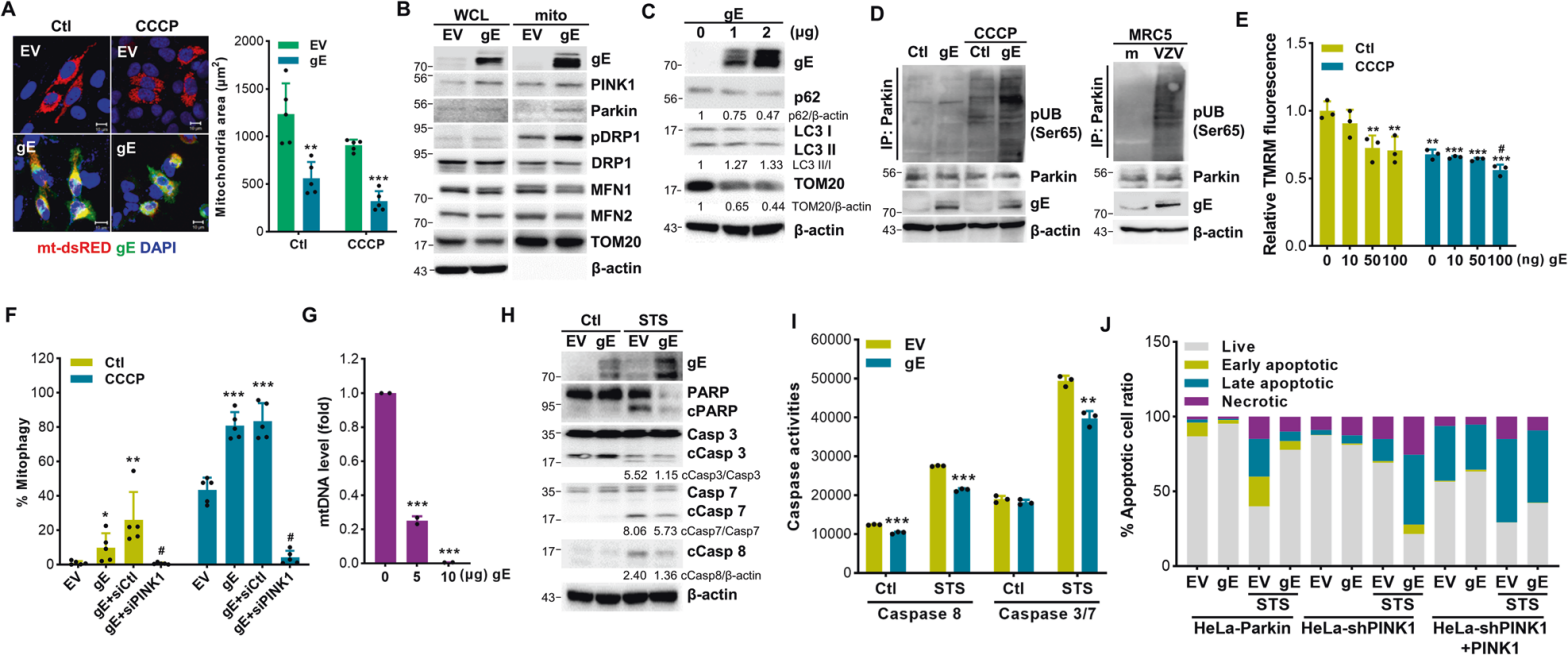
VZV gE modulates mitochondria dynamics and regulated PINK1/Parkin-dependent mitophagy. **A** HeLa-Parkin cells were transiently transfected with EV or VZV gE (green) along with mt-dsRED encoding plasmid (red) for 24 h and then treated with CCCP for 2 h. Quantification of the mitochondrial area is shown in the graph. **p < 0.01, ***p < 0.001 vs. EV-transfected cells. **B** HeLa-Parkin cells expressing either EV or VZV gE were subjected to immunoblot analysis. Whole-cell lysates (WCL) and mitochondrial fractions (mito) were isolated and VZV gE, PINK1, Parkin, phospho-DRP1(Ser616), DRP1, MFN1, MFN2, and TOM20 expression was measured. A representative of three independent experiments is shown. β-actin was used as a protein loading control. **C** p62, LC3, and TOM20 protein levels were examined in VZV gE transfection in HeLa-Parkin cells by immunoblot analysis. **D** HEK293T cells (Ctl) or HEK293T stably expressing VZV gE (gE) were treated with DMSO (Ctl) or CCCP for 2 h (left). MRC5 cells were infected with VZV (MOI 0.001) for 48 h (right). The ubiquitination of Parkin was measured by immunoblot assay using anti-phospho-ubiquitin (Ser65), Parkin, and VZV gE-specific antibodies. **E** Cells were transiently transfected with the indicated amount of VZV gE plasmid for 24 h and then treated with Ctl or CCCP for 2 h. Relative tetramethylrhodamine methyl ester (TMRM) intensities were measured. **p < 0.01; ***p < 0.001 vs. EV-transfected Ctl-treated cells. #p < 0.05 vs. VZV gE-transfected Ctl-treated cells. **F** HeLa-Parkin cells expressing mtKeima were transfected with EV or VZV gE plasmids along with siRNA control (siCtl) or siPINK1 (siPINK1) for 24 h. After CCCP treatment for 2 h, mitophagy levels were determined by confocal microscopy. *p < 0.05; **p < 0.01; ***p < 0.001 vs. EV-transfected cells. #*p* < 0.05 vs. gE-transfected siCtl-expressing cells. **G** Mitochondrial DNA (mtDNA) content of VZV gE-expressing HeLa-Parkin cells was analyzed by RT-qPCR (mean ± SD; *n* = 3). ***p < 0.001 vs. EV-transfected cells. **H** HeLa-Parkin cells were transiently transfected with EV or VZV gE plasmid; 24 h later, the cells were either stimulated with DMSO (Ctl) or 3 μM staurosporine (STS) for 2 h. Cleavage of PARP, caspase 3, 7, and 8 is shown by immunoblot analysis. β-actin was used as a protein loading control. Images are representatives of three independent experiments. **I** Activities of caspases 3/7 or caspase 8 in EV or VZV gE-expressing cells treated with STS was determined using caspase luminescence assays. Data represented as mean ± SD. **p < 0.01; ***p < 0.001 vs. EV-transfected cells in each group. **J** Flow cytometry analysis by Annexin V-FITC/PI staining showed % cells undergoing apoptosis in HeLa-Parkin, HeLa-shPINK1, and PINK1-V5-transfected HeLa-shPINK1 (HeLa-shPINK1 + PINK1) cells.

To understand the underlying mechanism of how VZV infection induces mitophagy, we next examined the role of VZV gE which is the most abundant glycoprotein. VZV gE is also known to manipulate early innate antiviral responses via IFN suppression [17]. HeLa cells stably expressing Parkin (HeLa-Parkin) cells were transfected with empty vector (EV) or VZV gE-specific plasmids with or without CCCP. Stable expression of Parkin in HeLa-Parkin cells was validated by immunoblot analysis (Fig. S3E). Figure 3A shows that VZV gE was localized in the mitochondria and led to enhanced mitochondrial fission upon CCCP treatment. In agreement with this, VZV gE led to decreased expression of mitochondrial fusion factors, such as MFN1 or MFN2; in contrast, it enhanced Ser616 phosphorylation of DRP1 in the mitochondria (Fig. 3B). Importantly, VZV gE led to Parkin recruitment to the mitochondria, suggesting a potential role for gE in modulating mitochondrial fission and mitophagy. We also examined whether VZV gE affects the expression of several ubiquitin-independent mitophagy receptors such as NDP52, OPTN, BNIP3, BNIP3L/NIX, however, no change in receptor expression occurred in VZV-gE-expressing cells (Fig. S4). Furthermore, VZV gE downregulated TOM20, increased LC3 II, and decreased p62 levels in a dose-dependent manner (Fig. 3C).

To further investigate whether VZV gE modulates PINK1/Parkin-dependent mitophagy, we also checked the ubiquitination level of Parkin which is catalyzed by PINK1. VZV infection or VZV gE expression triggered robust ubiquitination of Parkin, suggesting the importance of PINK1 and Parkin (Fig. 3D). In addition, VZV gE triggered mitochondrial depolarization and mitophagy, while PINK1 knockdown in HeLa-mtKeima cells inhibited VZV gE-induced mitophagy, suggesting that VZV gE specifically utilizes PINK1/Parkin-dependent mitophagy (Fig. 3E, F). In support of this, we also show that VZV gE failed to activate mitophagy in ATG5 knockout HeLa cells as shown in the unchanged expression levels of TOM20 and LC3-II (Fig. S5A). Along with genetic ablation of ATG5, chloroquine (CQ)-treated HEK293T cells stably expressing VZV gE showed a higher level of LC3 II compared to control cells, suggesting that VZV gE induced complete autophagy for lysosomal degradation of autophagosome (Fig. S5B, C). As a result of mitophagy, VZV gE overexpression led to a significant reduction in the quantity of mtDNA (Fig. 3G).

To gain additional insight into the effects exerted by gE, we evaluated whether gE could regulate apoptosis pathways. VZV gE overexpression showed impaired caspase activation in response to an apoptosis-stimulating drug, staurosporine (STS), as shown by immunoblotting and caspase activity assay results (Fig. 3H, I). The anti-apoptotic effect of VZV gE was further confirmed by flow cytometry using Annexin V/propidium iodide (PI) staining. A diminished number of cells undergoing STS-induced apoptosis was found in gE-expressing cells compared to EV-transfected cells (Fig. 3J). To determine whether mitophagy is responsible for VZV gE-driven protection against apoptosis, the same experiment was performed in HeLa cells stably expressing PINK1 shRNA (HeLa-shPINK1) cells. Notably, VZV gE failed to protect against STS-induced apoptosis by the loss of PINK1, whereas the anti-apoptotic function of gE was observed in PINK1-rescued cells, suggesting that VZV gE inhibited apoptosis via PINK1.

**Fig. 4:**
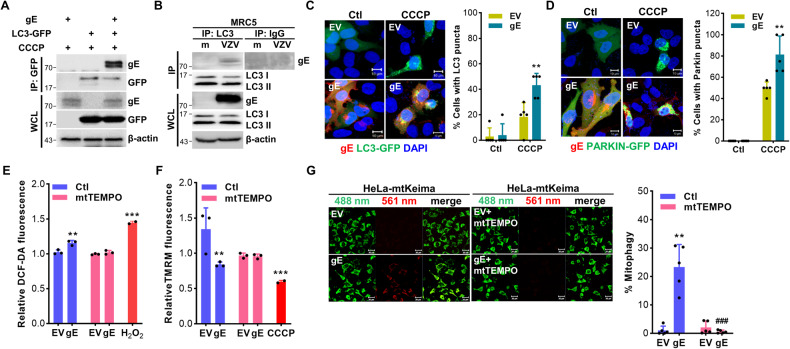
VZV gE regulates PINK1/Parkin-mediated mitophagy by interacting with LC3 and promoting mitochondrial reactive oxygen species production. **A** HeLa-Parkin cells were transfected with VZV gE and LC3-GFP-encoding plasmids in the presence of CCCP. **B** MRC5 cells were infected with VZV (MOI 0.001) for 48 h. Cells were lysed and precipitated using anti-GFP, or anti-LC3 antibodies. The whole-cell lysates (WCL) and immunoprecipitated proteins were analyzed using anti-VZV gE, anti-GFP, anti-LC3 antibodies. Anti-Rabbit immunoglobulin G (IgG) antibodies were used as a negative control at the endogenous level. β-actin was used as a protein loading control. **C**, **D** HeLa-Parkin cells were transfected with LC3-GFP (green) (**C**) or Parkin-GFP (green) (**D**) and VZV gE plasmids (red) and treated with control (Ctl) or CCCP for 2 h. The percentage (%) of cells with Parkin or LC3 puncta associated with gE is quantified and represented as a graph on the right. **p < 0.01 vs. EV-transfected CCCP-treated cells. **E** HeLa-Parkin cells were transfected with EV or VZV gE following treatment with or without 1 μM mitoTEMPO (mtTEMPO). After 24 h, DCF-DA fluorescence dye was added to the cells and fluorescence intensity was measured. 20 μM H_2_O_2_ was treated in cells for 2 h as a positive control. **p < 0.01; ***p < 0.001 vs. EV-transfected cells in Ctl groups. **F** Relative tetramethylrhodamine methyl ester (TMRM) intensities were measured. 20 μM CCCP treated in cells was used as a positive control. **p < 0.01; ***p < 0.001 vs. EV-transfected Ctl groups. **G** HeLa-Parkin cells expressing mtKeima were transfected with EV or VZV gE following treatment with or without 1 μM mtTEMPO for 24 h. Mitophagy levels were quantified. The bar graph shows mean ± SD from three experiments.

To delineate the molecular mechanisms by which VZV gE modulates mitophagy, we performed a co-immunoprecipitation (IP) assay. VZV gE interacted with LC3 in response to VZV infection and CCCP treatment (Fig. 4A, B). In correlation with VZV gE transient overexpression results, HEK293T stably expressing VZV gE also showed interaction with endogenous LC3 upon CCCP treatment but not in HEK293T or IP with IgG (Fig. S6A). VZV gE treatment led to a significant increase in the number of cells with LC3-GFP or Parkin-GFP puncta induced by CCCP treatment, suggesting that VZV gE promotes LC3 and Parkin activation, which eventually leads to mitochondrial degradation into lysosomes via mitophagy (Fig. 4C, D). Recent studies have suggested that mtROS production can contribute to mitophagy activation [18]. Thus, we evaluated whether mtROS production is upregulated upon VZV gE overexpression. Interestingly, VZV gE increased ROS production, whereas mtTEMPO, a mitochondria-targeted antioxidant, repressed VZV gE-induced ROS production (Fig. 4E). Furthermore, VZV gE-induced MMP depolarization and mitophagy was blocked by mtTEMPO (Fig. 4F, G). Taken together, these results suggest that VZV gE induces PINK1/Parkin-dependent mitophagy by interacting with LC3 and promoting mtROS production.

### VZV gE inhibits STING- and MAVS-mediated IFN pathways via mitophagy

**Fig. 5:**
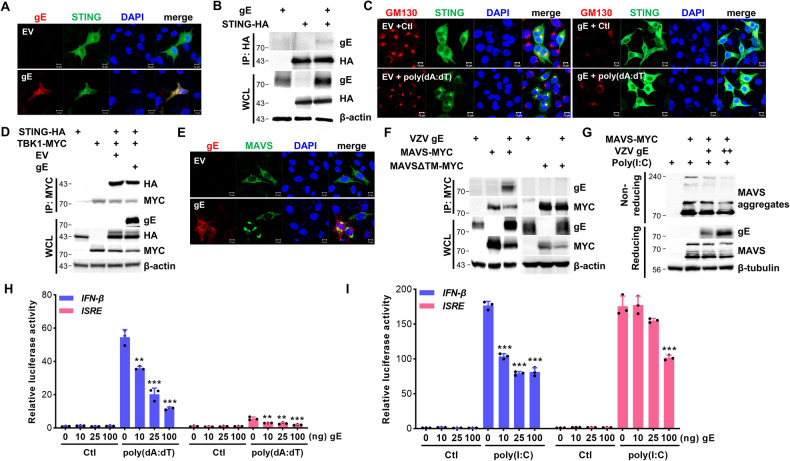
VZV gE antagonizes STING- and MAVS-mediated signaling pathways. **A** HeLa cells were transfected with EV or VZV gE (red) plasmid along with STING-HA (green). **B** HEK293T cells were transfected with VZV gE along with STING-HA-encoding plasmids. After 24 h, the whole-cell lysates (WCL) and immunoprecipitates were analyzed using anti-gE and anti-HA antibodies. β-actin was used as a protein loading control. **C** Confocal analysis of HeLa-Parkin cells transfected with either EV or gE plasmids followed by 0.5 μg/mL poly(dA:dT) treatment for 4 h. GM130 (red) staining for Golgi apparatus and STING (green) translocation is shown. **D** STING-HA and TBK1-MYC plasmid were transfected with EV or VZV gE in HEK293T cells. After 24 h of transfection, the cells were lysed and subjected to immunoprecipitation and immunoblot analysis against VZV gE, anti-HA, and anti-MYC tagging antibodies. β-actin was used as a protein loading control. **E** HeLa cells were transfected with EV or VZV gE (red) plasmid along with MAVS-MYC (green) plasmid. **F** HEK293T cells were transfected with VZV gE along with MAVS-MYC- or MAVSΔTM-MYC-encoding plasmids. After 24 h, the cells were lysed and precipitated using anti-MYC tag antibody. Whole-cell lysates (WCL) and immunoprecipitates were analyzed using anti-gE and anti-MYC antibodies. β-actin was used as a protein loading control. **G** HEK293T cells were transfected with VZV gE and MAVS-MYC-encoding plasmids for 24 h. Transfected cells were treated with DMSO or 20 μg/mL poly(I:C) for 2 h and the cell lysates were analyzed by non-reducing SDS-PAGE, to detect MAVS aggregates, and reducing SDS-PAGE to confirm the transfection efficiency. β-tubulin was used as a protein loading control. **H**, **I** Indicated amount of VZV gE with STING-HA (H) or MAVS-MYC (I) plasmid was transiently co-transfected with IFN-β or ISRE luciferase reporter plasmids. After 24 h of transfection, DMSO (Ctl) or 0.5 μg/mL poly(dA:dT) or 20 μg/mL poly(I:C) was added to the cells for 6 h. Relative luciferase activity is shown (mean ± SD; *n* = 3). **p < 0.01; ***p < 0.001 vs. EV-transfected cells.

Our previous study showed that STING is essential for the host defense mechanism against VZV infection in human dermal cells [17]. To investigate whether VZV gE affects the STING pathway, we performed co-IP experiments and showed that VZV gE specifically interacted with STING (Figs. 5A, B and S6B). In response to sensing viral DNA and/or cyclic dinucleotides, STING translocates from the endoplasmic reticulum (ER) to the Golgi apparatus (GA) and is associated with TBK1, which phosphorylates IRF3 to trigger IFN pathways [19]. As shown in Fig. 5C, EV-transfected cells showed STING translocation to the GA following poly(dA:dT) treatment, whereas VZV gE-expressing cells showed STING distribution in the cytoplasm, despite poly(dA:dT) treatment. We further investigated whether VZV gE affects STING/TBK1 interaction and Fig. 5D shows that VZV gE specifically disrupted the interaction between STING and TBK1.

MAVS acts as a critical adaptor protein that transduces antiviral signaling by physically interacting with RIG-I and MDA5 [20]. As expected, VZV gE colocalized and interacted with MAVS, as shown by confocal microscopy and immunoblot analyses (Figs. 5E, F and S6B). Notably, the transmembrane domain lacking MAVS (MAVSΔTM) failed to associate with VZV gE, suggesting a specific interaction between mitochondrial MAVS and VZV gE. Next, we tested whether VZV gE disrupts MAVS oligomerization upon poly(I:C) treatment. Figure 5G shows that VZV gE abrogated MAVS oligomerization in a dose-dependent manner. Finally, we also measured the effect of VZV gE overexpression on STING- or MAVS-induced signaling through IFN-β or ISRE promoter activation using a luciferase reporter assay. Figure 5H, I show that STING- or MAVS-mediated IFN-β and ISRE promoter activity was significantly reduced by VZV gE, emphasizing the role of VZV gE in counteracting multiple steps of the IFN-mediated antiviral pathways.

**Fig. 6:**
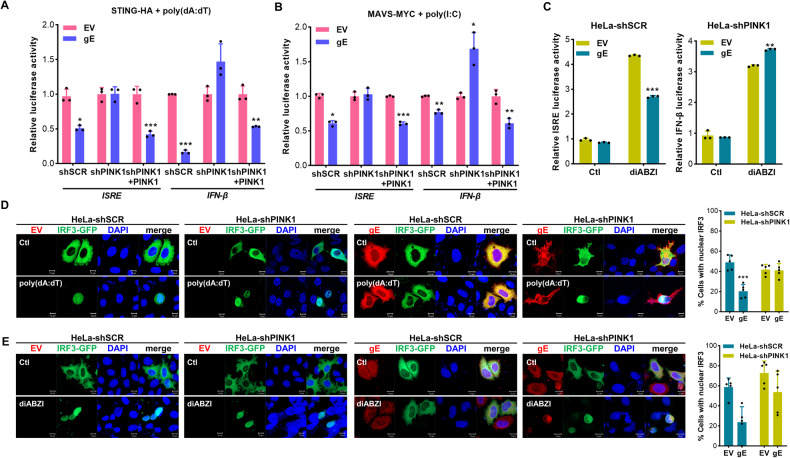
Mitophagy is required for gE-mediated inhibition of IFN production. **A**, **B** HeLa-shSCR (shSCR), HeLa-shPINK1 (shPINK1), PINK1-V5 transfected HeLa-shPINK1 cells (shPINK1 + PINK1) were transfected with EV, VZV gE, along with IFN-β or ISRE luciferase reporter plasmids. Cells were also co-transfected with STING-HA (**A**), MAVS-MYC (**B**) for 24 h. As indicated, the cells were treated with 0.5 μg/mL poly(dA:dT) or 20 μg/mL poly(I:C) for 6 h. Relative luciferase activity is shown (mean ± SD; *n* = 3). *p < 0.05; **p < 0.01; ***p < 0.001 vs. EV-transfected cells. **C** HeLa-shSCR and HeLa-shPINK1 cells were co-transfected with EV or VZV gE, STING-HA, IFN-β or ISRE luciferase reporter plasmids for 24 h; 10 μM diABZI was added to activate STING-mediated IFN-β or ISRE promotor. **p < 0.01; ***p < 0.001 vs. EV-transfected cells. **D**, **E** HeLa-shSCR or HeLa-shPINK1 cells were transfected with EV or VZV gE (red), along with IRF3-GFP plasmid (green). After 0.5 μg/mL poly(dA:dT) or 10 μM diABZI treatment, IRF3 nuclear translocation from cytoplasm was observed. For quantification of IRF3 nuclear localization, a minimum of one hundred cells per condition were counted in three independent experiments. Data represent the mean ± SD of three independent experiments. ***p < 0.001 vs. EV-transfected cells.

To determine if PINK1 is required for gE-mediated inhibition of IFN production, we performed an ISRE or IFN-β luciferase reporter assay in HeLa-shPINK1 cells. VZV gE significantly inhibited STING- or MAVS-mediated IFN-β and ISRE promotor activities in HeLa-shSCR cells but not in the absence of PINK1 (Fig. 6A, B). Furthermore, VZV gE suppressed IFN-β and ISRE promoter activation in response to STING agonists (diABZI) but this effect was not observed in HeLa-shPINK1 cells (Fig. 6C). Next, we examined whether VZV gE attenuated IRF3 activation via mitophagy. In response to poly(dA:dT) stimulation, IRF3 translocated to the nucleus; however, IRF3 failed to translocate to the nucleus in the presence of gE (Fig. 6D). Figure 6E shows that VZV gE blocked IRF3 nuclear translocation in response to diABZI, but only in the presence of PINK1. Therefore, mitophagy is essential for VZV gE to abrogate STING- and MAVS-mediated pathways.

### VZV-mediated mitophagy modulates antiviral immunity in the human SOC model

**Fig. 7:**
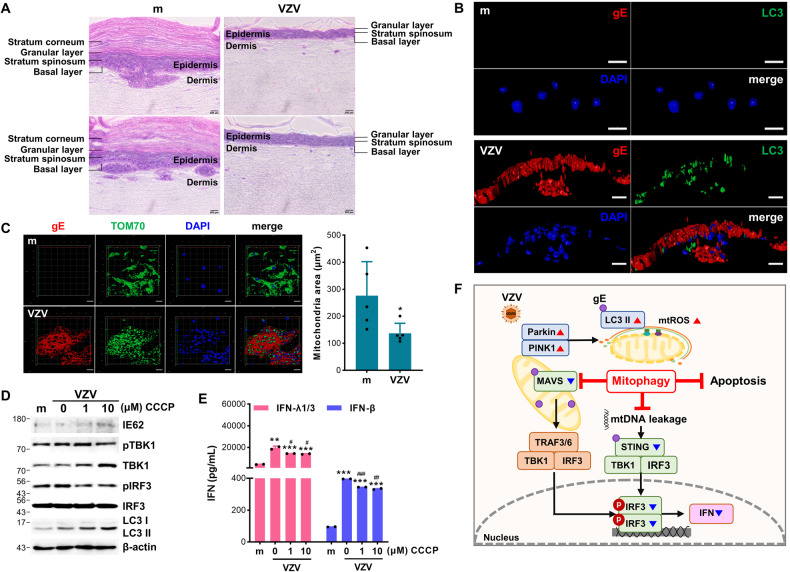
VZV infection triggers mitochondrial fission and CCCP suppresses STING-mediated IFN production in human skin organ culture (SOC). **A** The human epidermal layer of SOC was exposed to 10^5^ PFU cell-free VZV (MG1111) for 7 days and subjected to hematoxylin and eosin staining. **B**, **C** Mock (m) or VZV-infected SOC was subjected to immunofluorescence staining against VZV gE (red) and LC3 (green) or TOM70 (green). Quantification of the mitochondrial area is shown in the graph. **D** Human epidermal layer of SOC was treated with the indicated concentration of CCCP and infected with VZV. At 7 days post-infection, proteins from SOC were harvested and VZV IE62, phospho-TBK1, TBK1, phospho-IRF3, IRF3, and LC3 protein expression levels were measured by immunoblot analysis. β-actin was used as a protein loading control. **E** Conditioned media were collected and secretion of type I (IFN-β) and III (IFN-λ1/3) IFN levels was measured by ELISA. **p < 0.01; ***p < 0.001 vs. mock-infected groups. #*p* < 0.05; ##*p* < 0.01; ###*p* < 0.001 vs. VZV-infected group without CCCP treatment. **F** A graphical illustration of VZV gE-mediated regulation of mitophagy is shown.

Given that the SOC model has been used for the characterization of autophagy during VZV infection [21], we utilized the human SOC model to examine the effects of mitophagy during VZV infection. The differentiated epidermis was exposed to cell-free VZV for seven days. Hematoxylin and eosin staining analysis showed that VZV infection led to separation of the epidermis, including the stratum corneum and granular layer, and reduction of epidermal thickness compared with mock-infected SOC (Fig. 7A). Furthermore, VZV gE was detected mainly in the epidermal layer, and there were multiple syncytial formations, which were recognized as multinucleated cells and were formed due to VZV infection [22]. Along with histopathological changes caused by VZV infection, LC3 puncta formation was detected only in the VZV gE-positive region, while the mitochondrial area, which was quantified as shown in the graph, was diminished compared to that in the mock group (Fig. 7B, C). Similar to our in vitro cell culture results, there was an increased expression level of LC3-II following VZV infection, and CCCP treatment upregulated VZV IE62 protein expression in the SOC model. Furthermore, CCCP treatment in the SOC model significantly attenuated TBK1/IRF3 activation and IFN secretion in response to VZV infection (Fig. 7D, E).

## Discussion

In this study, we discovered a novel role for VZV gE in disrupting mitochondrial dynamics via triggering mitochondrial fission and Parkin recruitment into the mitochondria. Our data reveal molecular insights into how VZV gE manipulates PINK1/Parkin-mediated mitophagy to evade the host’s innate immune response and enhance viral infection in human skin models. We suggest that PINK1/Parkin-mediated mitophagy is a novel strategy for the immune evasion mechanisms utilized by VZV, graphically illustrated in Fig. 7F.

Multiple groups have reported that viruses hijack mitophagy to favor their replication and manipulate the immune response in various ways. For example, coxsackievirus B3 infection disrupts IFN responses via mitophagy activation in human neural progenitor cells and rat cardiomyocytes [16] and vesicular stomatitis virus (VSV) infection induces RNF34-mediated mitophagy to lead to autophagic degradation of MAVS [23]. Similar to VSV, NS1 and PB1-F2 proteins of influenza viruses can modulate mitophagy, and in particular, PB1-F2 specifically interacts with TUFM to inhibit the innate immune response [24, 25], whereas hantavirus glycoprotein translocates to the mitochondria, interacts with TUFM, recruits LC3, and promotes mitophagy for MAVS degradation and IFN suppression [26]. As for herpesvirus, human herpesvirus-8 vIRF-1 activates NIX-mediated mitophagy during lytic replication [27] and Epstein–Barr virus-encoded BHRF1 contributes to the induction of PINK1/Parkin-dependent mitophagy to abolish the MAVS-mediated type I IFN response [28]. Given that VZV does not encode homologs of herpes simplex virus-1 (HSV-1) autophagy-inhibiting proteins, such as ICP34.5 and US11 [10, 29], it is plausible that VZV promotes the autophagy process differently from that by HSV-1. It will be worth investigating in the future if other VZV proteins contribute to the alteration of mitochondrial quality control mechanisms via mitophagy.

It has been previously demonstrated that VZV infection triggers autophagy, especially in human skin cells, SOC, and skin biopsies of patients with zoster. Takahashi et al. demonstrated that regardless of a cell-associated or cell-free virus, VZV-infected MRC5, keratinocytes, or melanocytes show autophagosome formation [8] and Buckingham et al. showed that VZV infection induces complete autophagy, which results in the degradation of the autophagosome by fusion with the lysosome [10]. Consistently, we confirmed that VZV infection of the skin induces autophagosome formation and mitophagy activation, using both skin cell and organ culture models. Additionally, our data demonstrated that the modulation of autophagy via chemical drugs can manipulate VZV infectivity and glycoprotein synthesis.

Here, we report for the first time that VZV gE-mediated mitophagy dampens IFN pathways by promoting the fragmentation and subsequent sequestration of mitochondria in autophagosomes. Given that VZV gE was found to interact with LC3, it will be intriguing to further investigate which domains of VZV gE bind the autophagosome adapter LC3 through an LC3 interacting region. Our results also highlight the ability of gE to translocate to the mitochondria and interact with LC3 to increase mitophagy, and operate as an apoptotic suppressor. Notably, multiple ORFs encoded by VZV were reported to have anti-apoptotic mechanisms, demonstrating the importance of modulating apoptosis for viral pathogenesis [30–32]. Mitochondria orchestrate diverse cell death processes in response to various stimuli and virus-induced mitophagy can attenuate apoptosis and increase viral persistence [3]. For instance, hepatitis B and C viruses disrupt mitochondrial dynamics and subsequently trigger mitophagy, but inhibit virus-induced apoptosis for viral persistence and production [33, 34]. Similar to these viruses, our results showed that VZV gE inhibited STS-induced apoptosis, whereas VZV gE failed to inhibit apoptosis in PINK1-knockdown cells. Together with the anti-apoptotic role of VZV gE, our data highlighted that VZV gE is likely to manipulate mitophagy to inhibit the host’s innate antiviral response.

It is commonly known that excessive ROS production from mitochondria has a close link with mitophagy [1, 18]; on the contrary, Parkin suppresses mtROS production and NLRP3-mediated inflammation [35]. Several viral proteins, such as NS5A encoded by the hepatitis C and classical swine fever viruses, trigger ROS production, causing PINK1/Parkin-dependent mitophagy for viral replication [36, 37]. These insights suggest the possibility that mtROS production triggers mitochondrial damage and promotes mitochondrial fragmentation in VZV-infected cells. In addition to mtROS, mtDNA leakage can trigger STING-mediated inflammation and IFN pathways [38, 39]. In support of these reports, we observed that gE-mediated mitophagy suppressed STING-mediated IFN production and IFN-induced gene expression, and PINK1-mediated mitophagy restrained VZV-induced mtDNA release into the cytosol to alter the STING pathway. Thus, these data suggest that mitochondrial dynamics and mitophagy are likely to be key regulators of STING pathways. Meanwhile, gE also counteracted MAVS-mediated IFN activation. Considering that MAVS facilitates the recruitment of NLRP3 to the mitochondria to upregulate its oligomerization and activation [20] and VZV infection triggers activation of NLRP3 inflammasome [40], the potential link between mitophagy and NLRP3 inflammasome should be investigated further.

In conclusion, this study sheds new light on mitophagy function as a viral immune evasion mechanism. Our data highlight the importance of VZV gE in the regulation of host immunity and introduce a mechanism by which viruses interfere with innate immune signaling via mitophagy. Taken together, our study uncovered molecular mechanisms underlying VZV-mediated manipulation of mitochondrial quality control and identified important regulators of VZV-induced mitophagy that may lead to the discovery of novel therapeutic antiviral targets.

## Materials and methods

### Cells, viruses, and reagents

HeLa, HEK293T, human fetal lung fibroblasts (MRC5), and immortalized human keratinocytes (HaCaT) cells were obtained from the American Type Culture Collection and cultured in DMEM (GenDEPOT, Katy, TX, USA) supplemented with 10% fetal bovine serum (FBS; (GenDEPOT) and 1% penicillin/streptomycin (Sigma-Aldrich, St. Louis, MO, USA). THP-1 cells were maintained in RPMI 1640 (Hyclone, Logan, UT, USA) supplemented with 10% FBS, 2 mM glutamine, 10 mM HEPES, 1 mM sodium pyruvate, 0.5 mM 2-mercaptoethanol, and 1% penicillin/streptomycin. Scrambled shRNA (shSCR) and DRP1-specific shRNA-expressing (shDRP1) THP-1 cells have been described previously [16, 41]. HeLa cells stably expressing ATG5 knockout, PINK1-specific shRNA, Parkin, and mitochondria-targeted fluorescent protein Keima (mtKeima) were generated as previously described [16, 42]. HEK293T cells stably expressing VZV gE were generated for this study.

VZV YC01 (GenBank Accession No. KJ808816) was propagated in MRC5 cells as previously described, and the virus titer was measured using a plaque assay as previously described [43]. Cell-free VZV strain, MG1111 (MAV/06), was obtained from GC Biopharma (Yongin, Korea).

CCCP, mtTEMPO, H_2_O_2_, and CQ were purchased from Sigma-Aldrich. Poly(dA:dT), poly(I:C), and diABZI were purchased from InvivoGen (San Diego, CA, USA). Staurosporine was purchased from Tocris (Bristol, UK).

### Plasmids, siRNA, and transfection

The following plasmids were used for this study; mito-dsRED, Parkin-GFP, LC3-GFP, pUNO-STING-HA, pEGFP-IRF3, pcDNA3-MAVS-MYC, or pcDNA3-MAVS-ΔTM-MYC, PINK1-V5, Parkin-MYC, pTargeT-VZV gE (a gift from Charles Grose; Addgene plasmid # 60845) [9]. For RNA interference, cells were seeded in 6-well plates and cultured until 70% confluency on the day of transfection. Transient transfections using control scrambled, PINK1, or Parkin-specific siRNAs (Bioneer, Korea) were performed using the RNAiMAX transfection reagent (Invitrogen) according to the manufacturer’s protocol. To recover the expression of PINK1 or Parkin, PINK1 or Parkin-specific siRNA-transfected cells were transfected with PINK1-V5 or Parkin-MYC plasmids using Lipofectamine 2000 (Invitrogen) for further experiments.

### Transmission electron microscopy

Mock or VZV-infected MRC5 cells were pre-fixed with PBS containing 2.5% glutaraldehyde for 4 h at 4 °C. The fixed cells were washed three times with PBS and then post-fixed with reduced osmium tetroxide for 1.5 h at 4 °C. After three times washing with PBS, post-fixed cells were dehydrated by a graded series of ethanol and embedded in Epon resin. Samples were prepared into ultra-thin sections (70–80 nm), and sections were stained with uranyl acetate and lead nitrate. Images were captured using the Hitachi H-7000 electron microscope (Hitachi, Japan).

### Immunofluorescence assay

Cells were seeded in the confocal dishes with different treatments, fixed with 4% paraformaldehyde, and permeabilized with 0.1% Triton X-100 diluted in PBS. Next, the cells were blocked with 2.5% bovine serum albumin (BSA) in PBS for 20 min. Primary antibodies against VZV gE (SC-56995), GM130 (BD Biosciences; 610822), anti-HA (51064-2-AP), and anti-MYC (16286-1-AP) were diluted in 0.5% BSA in PBS and incubated with cells for 1 h, followed by secondary antibody incubation using anti-mouse Alexa 594 (Invitrogen; A11005), anti-mouse FITC (Invitrogen; 31569) and anti-rabbit Alexa 488 (Invitrogen; A11008). The cells were then stained with 4ʹ,6-diamidino-2-phenylindole (DAPI).

To measure mitolysosome formation, the cells were treated with media containing MitoTracker Green (Invitrogen) and LysoTracker Deep Red (Invitrogen) to visualize mitochondria and lysosomes. After 30 min, the cells were imaged using a confocal microscope (LSM900; Carl Zeiss) and the percentage of cells with mitolysosomes was quantified by counting more than 100 cells. The quantification of mitophagy was assessed by measuring pH-dependent fluorescence using HeLa-mtKeima cells, as previously described [16, 24].

### MMP and ROS measurement

To measure MMP, cells were seeded in 96-well black plates and infected with VZV or transfected with VZV gE-encoding plasmids. At the desired time point, the cells were incubated in serum-free media supplemented with 200 nM TMRM (Thermo Fisher Scientific) and incubated at 37 °C for 30 min. For the detection of ROS, cells were stained with 10 μM 2′,7′-dichlorofluorescein diacetate (DCF-DA; Sigma-Aldrich) in serum-free media for 30 min. The fluorescence intensity was measured using a Varioskan LUX multimode microplate reader (Thermo Fisher Scientific).

### Real-time quantitative polymerase chain reaction (RT-qPCR)

Total RNA was isolated from the cells using a DirectZol RNA kit (Zymo Research). An aliquot of 1 μg total RNA was reversely transcribed using the ImProm-II Reverse Transcription System (Promega) according to the manufacturer’s instructions. Quantitative PCR was performed in triplicates with the Power SYBR® Green Master Mix (Invitrogen). Gene expression levels were examined by QuantStudio 6 Flex Real-time PCR system (Thermo Fisher Scientific) under the following conditions: 95 °C for 10 min, followed by 40 cycles at 95 °C for 30 s and 60 °C for 1 min. Primer sequences were described in previous studies [17, 44]. Relative mRNA levels were calculated based on the 2^−ΔΔCt^ method. For quantification of mitochondrial DNA, gDNA was isolated from the cells and RT-qPCR was performed as previously described [45].

### Sodium dodecyl sulfate-polyacrylamide gel electrophoresis (SDS-PAGE)/non-reducing SDS-PAGE immunoblot analysis

Cells were lysed in ice-cold radioimmunoprecipitation assay (RIPA) buffer (Sigma-Aldrich) containing protease and phosphatase inhibitors (Roche), and SDS-PAGE was performed as previously described [16]. For SDS-PAGE, samples were prepared in a solution containing 50 mM Tris-HCI pH 6.8, 1% 2-mercaptoethanol, 2% SDS, 0.1% bromophenol blue, and 10% glycerol and boiled at 95 °C for 15 min. For non-reducing SDS-PAGE, samples were prepared in a solution containing 30 mM Tris-HCI pH 6.8, 1% SDS, and 0.1% bromophenol blue, and 10% glycerol without boiling. Prepared proteins were separated, transferred onto polyvinylidene difluoride (PVDF) membranes, and blocked with 5% skim milk in Tris-buffered saline (TBS) supplemented with 0.1% Tween-20 (TBS/Tw) for 1 h. The membranes were then incubated with primary antibodies at 4 °C overnight. The following antibodies were used; VZV gE (SC-56995), TOM20 (SC-11415), VZV IE62 (SC-17525), and anti-GFP (SC-8334) were purchased from Santa Cruz; ATG5 (CST#12994), p62 (CST#8025), pDRP1 (CST#4494), DRP1 (CST#9570), MFN1 (CST#14739), MFN2 (CST#11925), Parkin (CST#4211), pTBK1 (CST#5483), STING (CST#13647), TBK1 (CST#3504), pIRF3 (CST#29047), IRF3 (CST#11904), PARP (CST#9542), caspase 3 (CST#9662), caspase 7 (CST#9492), cleaved caspase 8 (CST#9496), Phospho-Ubiquitin (Ser65) (CST# 62802) and anti-HA (CST#2367) were obtained from Cell Signaling Technology; LC3 (NB100-2220) was purchased from Novus and β-actin (AM1021B) was purchased from Abcepta; PINK1 (23274-1-AP), NDP52 (CST#60732), OPTN (CST#58981), BNIP3 (CST#44060), BNIP3L/NIX (CST#12396),β-Tubulin (66375-1-lg), and anti-MYC (16296-1-AP) were purchased from ProteinTech; MAVS (A300-782A) was purchased from Fortis Life Science. Densitometry was performed to quantify the protein bands using ImageJ software, and a representative image of three independent experiments is shown.

### Co-IP

Cells were lysed in immunoprecipitation lysis buffer (Thermo Fisher Scientific) supplemented with a complete protease inhibitor cocktail (Roche). Co-IP was performed using the SureBeads Protein A magnetic beads (Bio-Rad Laboratories) according to the manufacturer’s instructions. SureBeads were conjugated with 1 μg antibodies for 15 min. The following antibodies were used; anti-GFP (SC-8334) from Santa Cruz Biotechnology, anti-HA (51064-2-AP) and anti-MYC (16286-1-AP) from Proteintech, anti-STING (CST#13647) from Cell Signaling Technology, anti-MAVS (A300-782A) was purchased from Fortis Life Science and anti-LC3 (NB100-2220) from Novus. After antibody conjugation, 500 μg cell lysate was added, and incubated overnight at 4 °C. Immunoprecipitates were washed with 0.1% PBS/Tw three times, and proteins were eluted in SDS sample buffer and analyzed by immunoblot anaylsis to detect endogenous interactions between proteins. To examine ubiquitinated Parkin, lysed cells were incubated with magnetic beads which were conjugated with Parkin antibody (CST#4211) for 24 h and then immunoprecipitates were analyzed by immunoblot assay using phospho-ubiquitin (CST#62802).

### Luciferase reporter assay

Luciferase reporter assay was performed as previously described [44]. Briefly, HeLa or HeLa-shPINK1 cells were seeded in a 96-well plate with 2% DMEM and transiently transfected with 50 ng of IFN-β or ISRE luciferase reporter plasmids in combination with STING-HA or MAVS-MYC with or without PINK1-V5 or VZV gE-encoding plasmid. The total amount of DNA was consistent with the addition of the appropriate EV. After 24 h, cells were treated with diABZI, poly(I:C), or poly(dA:dT) to stimulate IFN-β or ISRE promoter. Dual-Glo luciferase reporter assay system (Promega) was used to lyse the cells and measure the luciferase activity.

### Enzyme-linked immunosorbent assay (ELISA)

The conditioned media were collected to examine cytokine and IFN secretion. IL-6, IL-8, IFN-β, and IFN-λ1/3 secretion levels were measured by ELISA kit (R&D Systems) following the manufacturer’s instructions.

### Cell death and viability assay

For measurement of caspase activity, HeLa-Parkin cells were transfected with EV or VZV gE and treated with 3 μM staurosporine for 2 h. A Caspase-Glo assay kit (Promega) was used according to the manufacturer’s instructions. Luminescence was measured using a Varioskan LUX Multimode Microplate Reader (Thermo Fisher Scientific). To measure apoptotic cell death, cells were harvested and stained using a fluorescein isothiocyanate (FITC)-conjugated Annexin V Apoptosis Detection Kit (BD Biosciences) according to the manufacturer’s instructions. The stained cells were analyzed using a BD LSR Fortessa X-20 flow cytometer (BD Biosciences). To examine cell cytotoxicity in CCCP-treated MRC5 and HaCaT cells, a CCK-8 (Dojindo, USA) assay was performed according to the manufacturer’s instructions.

### Human SOC model

KeraSkin-FT^TM^ (BioSolution, Korea), a human 3D epidermal and dermal SOC, was constructed as previously described [46]. After differentiation, the epidermal layer was exposed to mock or cell-free VZV (MG1111, 10^5^ plaque-forming unit (PFU) for 7 days with or without CCCP treatment. To validate the multilayered and fully differentiated skin model, SOC was examined by H&E staining. In detail, SOC sections were dehydrated using a stepwise ethanol gradient (95%, 95%, 100%, 100%, xylene, and xylene) and embedded in paraffin. For immunofluorescence staining, SOC sections were rehydrated using ethanol gradient (xylene, xylene, 100%, 100%, 90%, 80%, and 70%) and boiled in antigen retrieval buffer (pH 6.0) for 15 min. Slides were permeabilized, blocked, and immunostained with primary antibodies against VZV gE (SC-56995), LC3 (CST #3868), and TOM70 (14528-1-AP).

### Statistical analysis

Data are presented as the mean ± standard deviation (SD) from at least three independent experiments. Statistical comparisons between different treatments were performed using the Mann–Whitney *U*-test or Student’s t-test, and results were considered statistically significant at p < 0.05. All statistical analyses were performed using GraphPad Prism 9 software.

### Reporting summary

Further information on research design is available in the Nature Research Reporting Summary linked to this article.

### Supplementary information

Reporting Summary

Supplementary information

Original Data File

## Data Availability

The data that support the findings of this study are available from the corresponding author upon reasonable request.
