# Supplementary material for: Varicella zoster virus glycoprotein E facilitates PINK1/Parkin-mediated mitophagy to evade STING and MAVS-mediated antiviral innate immunity
Source: Cell Death Dis. 2024 Jan 6;15(1):16. doi: 10.1038/s41419-023-06400-z (PMC10771418; doi:10.1038/s41419-023-06400-z)

**Supplementary information**

**Fig S1. VZV infection leads to formation of double-membrane vesicles containing mitochondria**

Ultrastructure of double-membrane vesicles containing mitochondria in VZV-infected MRC5 cells was observed by transmission electron microscopy. Red arrows indicate double-membrane vesicles containing mitochondria. Scale bar = 1μM


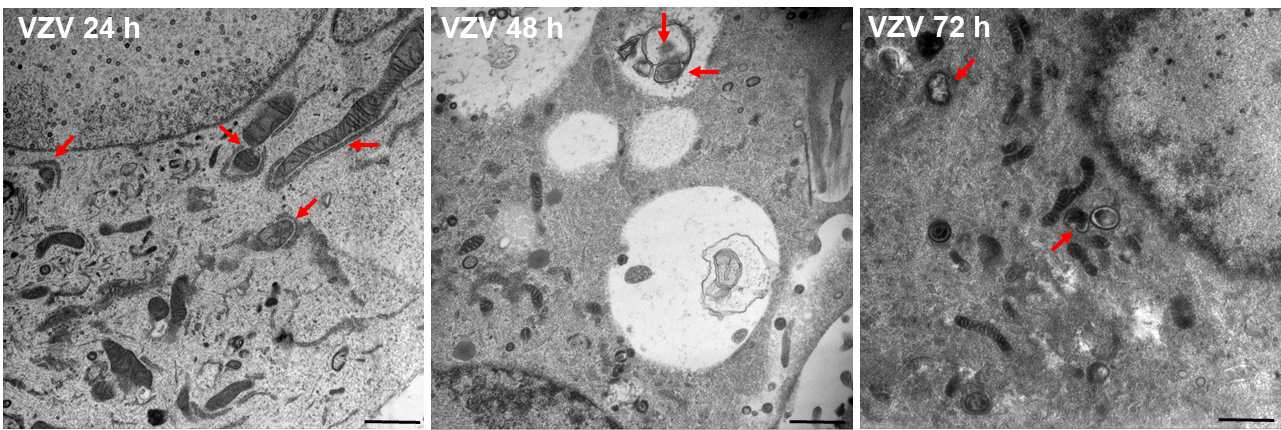


**Fig S2. Effect of CCCP on mitolysosome formation and cell viability**

(A) MRC5 and HaCaT cells were treated with CCCP for 2 h and stained with MitoTracker (green) and LysoTracker (red). Confocal images were captured to show the formation of mitolysosomes. (B) MRC5 and HaCaT cells were treated with different concentrations of CCCP for 2 h and cell viability was measured via Cell Counting Kit-8 (CCK-8) assay.


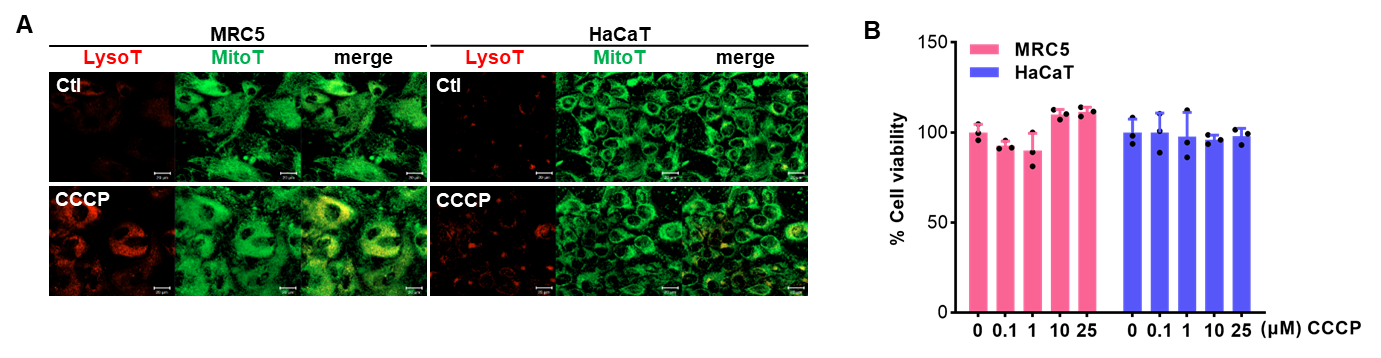


**Fig S3. Confirmation of PINK1 and Parkin overexpression or knockdown in HaCaT and HeLa cells**

(A) HaCaT cells were transfected with control (siCtl) or PINK1-specific siRNA (siPINK1) and incubated for indicated time points. RNA was extracted from cells to analyze PINK1 gene expression by qRT-PCR. ***p < 0.001 vs. siCtl-transfected cells at the indicated time points. hours post transfection; hpt (B) Control or PINK1-specific siRNA transfected HaCaT cells were infected with VZV (MOI 0.001). At desired time point, RNA was extracted from cells to analyze PINK1 gene expression by qRT-PCR. **p < 0.01, ***p < 0.001 vs. siCtl-transfected VZV-infected cells at the indicated time points. hours post infection; hpi (C, D) HaCaT cells were transiently transfected with control, PINK1, or Parkin-specific siRNA for knockdown. After 24 h, cell was transfected with PINK1-V5 or Parkin-MYC plasmids to recover PINK1 or Parkin expression. To confirm the overexpression or knockdown efficiency of PINK1 or Parkin, cell lysates were analyzed by immunoblot assay. β-actin was used as a protein loading control. (E) HeLa and HeLa stably expressing Parkin (HeLa-Parkin) cells were analyzed by immunoblot assay to examine stable protein expression of Parkin. β-actin was used as a protein loading control.


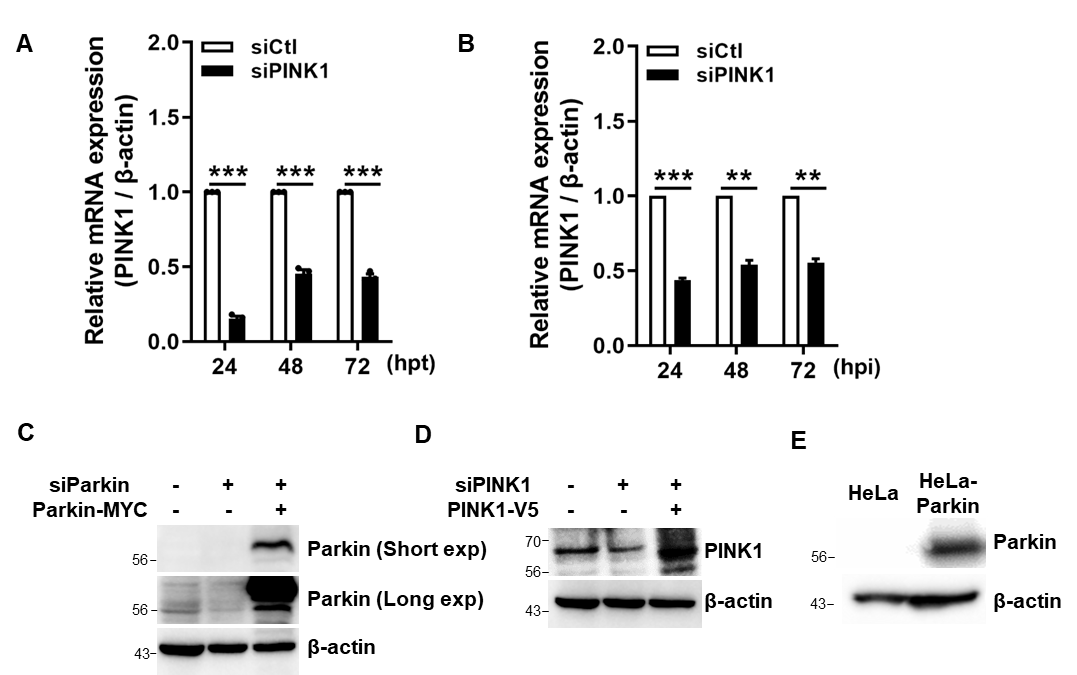


**Fig S4. Expression of mitophagy receptors in VZV gE-expressing cells**

Cells were transiently transfected with EV or VZV gE-encoding plasmid. After 24 h, VZV gE, NDP52, OPTN, BNIP3, BNIP3/NIX, p62 and LC3 was analyzed by immunoblot assay.


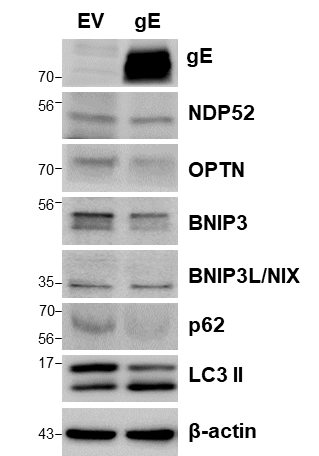


**Fig S5. Increased autophagy flux in VZV gE-expressing cells**

(A)ATG5 knockout HeLa cells (HeLa-ATG5 KO) were transiently transfected with VZV gE plasmid followed by 10 μM chloroquine (CQ) treatment for 6 h. Protein expression of VZV gE, ATG5, p62, LC3, and TOM20 was analyzed by immunoblot assay. β-actin was used as a protein loading control. (B) HEK293T cells and HEK293T cells stably expressing gE (HEK293T-gE) were treated with 10 μM CCCP for 2 h together with 10 μM CQ. VZV gE and LC3 protein expression levels were measured. β-actin was used as a protein loading control. (C) Cells were transiently transfected with EV or VZV gE plasmid and incubated for 24 h and then treated with or without negative control (Ctl), 10 μM of CQ for 4 h before harvesting cells for immunoblotting against VZV gE and LC3. β-actin was used as a protein loading control. The images are representative of three independent experiments.


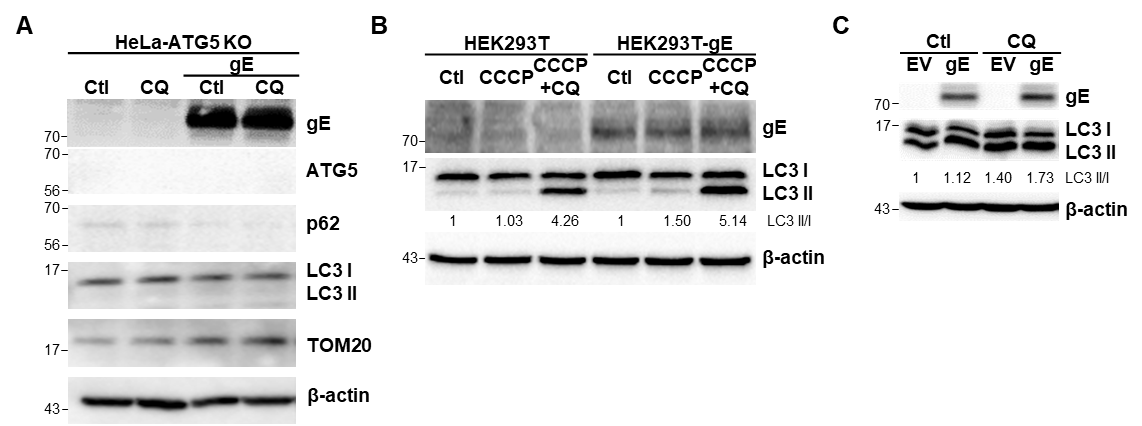


**Fig S6. Interaction of VZV gE with LC3, STING and MAVS at endogenous level**

(A, B) HEK293T or HEK293T stably VZV gE expressing cells (HEK293T-gE) were treated with 20 μM CCCP for 2 h. Cells were lysed and precipitated proteins were analyzed. Anti-Rabbit immunoglobulin G (IgG) antibodies were used as a negative control at endogenous level. β-actin was used as a protein loading control. Red asterisks represent VZV gE specific band (~78 kDa).


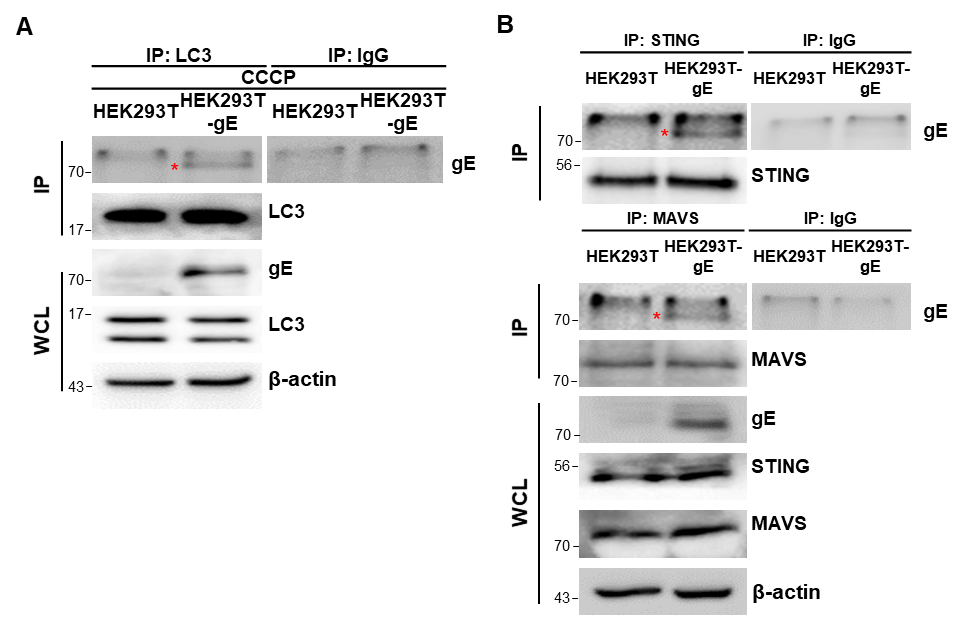

Supplement: Supplementary file 2 — Supplementary information [file 41419_2023_6400_MOESM2_ESM.docx]
