# Supplementary material for: Varicella zoster virus glycoprotein E facilitates PINK1/Parkin-mediated mitophagy to evade STING and MAVS-mediated antiviral innate immunity
Source: Cell Death Dis. 2024 Jan 6;15(1):16. doi: 10.1038/s41419-023-06400-z (PMC10771418; doi:10.1038/s41419-023-06400-z)
Supplement: Supplementary file 3 — Original Data File [file 41419_2023_6400_MOESM3_ESM.pdf]

FIG1D

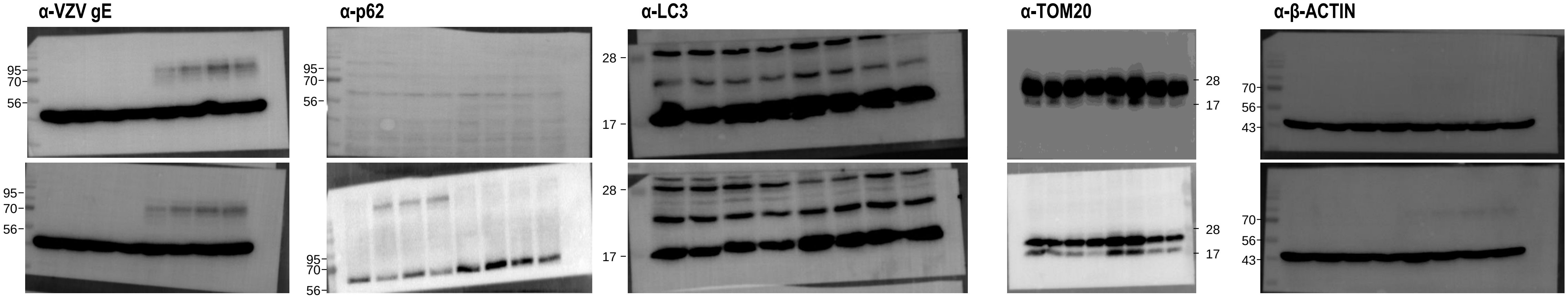
α-VZV gE
α-p62
α-LC3
α-TOM20
α-β-ACTIN
95
70
56
28
17
43

FIG2B

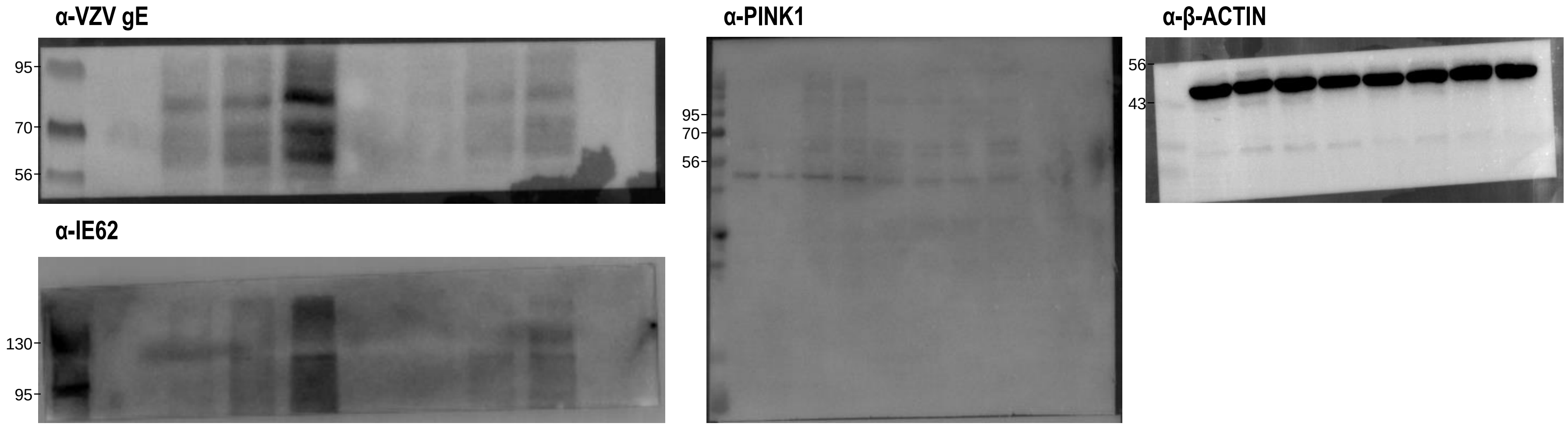
α-VZV gE
α-PINK1
α-β-ACTIN
α-IE62
95
70
56
43
130

FIG2D

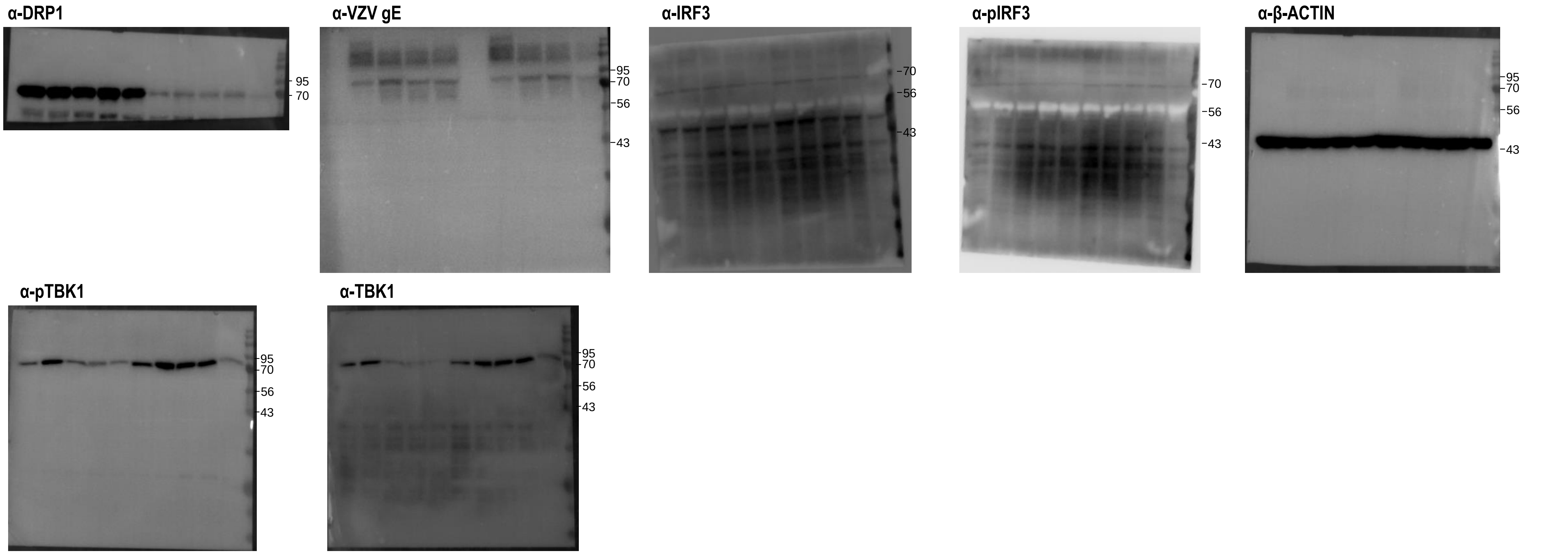

α-DRP1
95
70
α-VZV gE
95
70
56
43
α-IRF3
70
56
43
α-pIRF3
70
56
43
α-β-ACTIN
95
70
56
43
α-pTBK1
95
70
56
43
α-TBK1
95
70
56
43

FIG2I

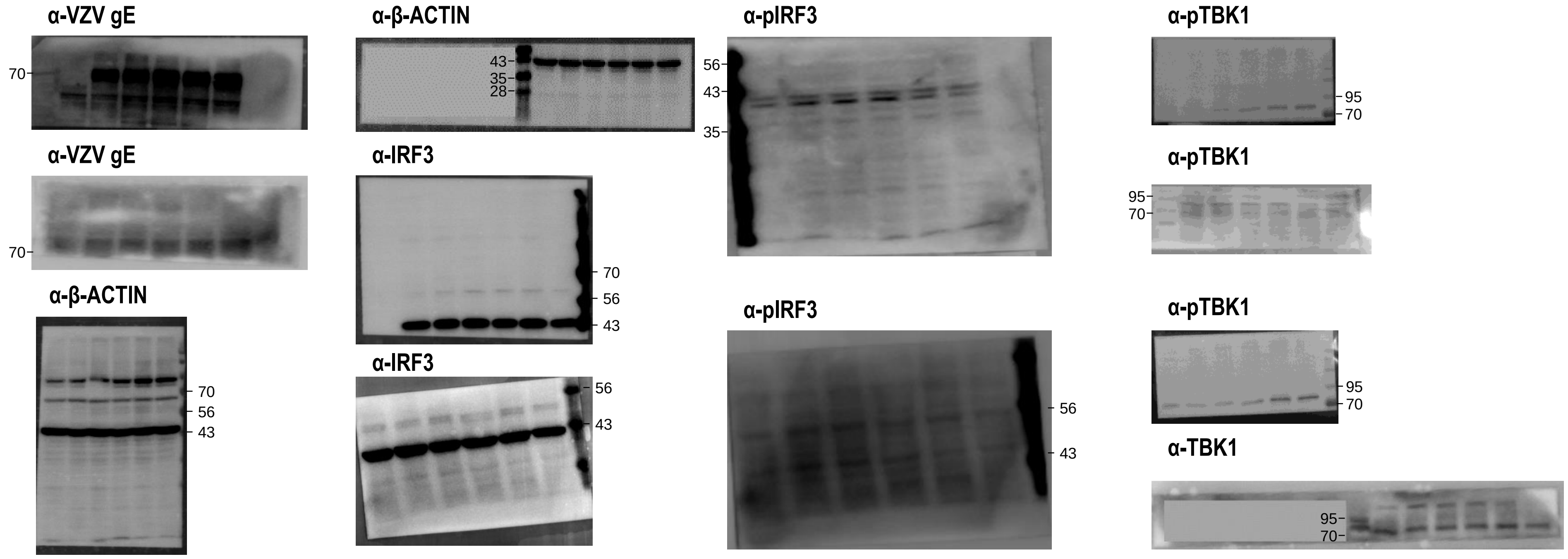
α-VZV gE
70
α-VZV gE
70
α-β-ACTIN
70
56
43
α-β-ACTIN
43
35
28
α-IRF3
70
56
43
α-IRF3
56
43
α-pIRF3
56
43
35
α-pIRF3
56
43
α-pTBK1
95
70
α-pTBK1
95
70
α-pTBK1
95
70
α-TBK1
95
70

FIG3B

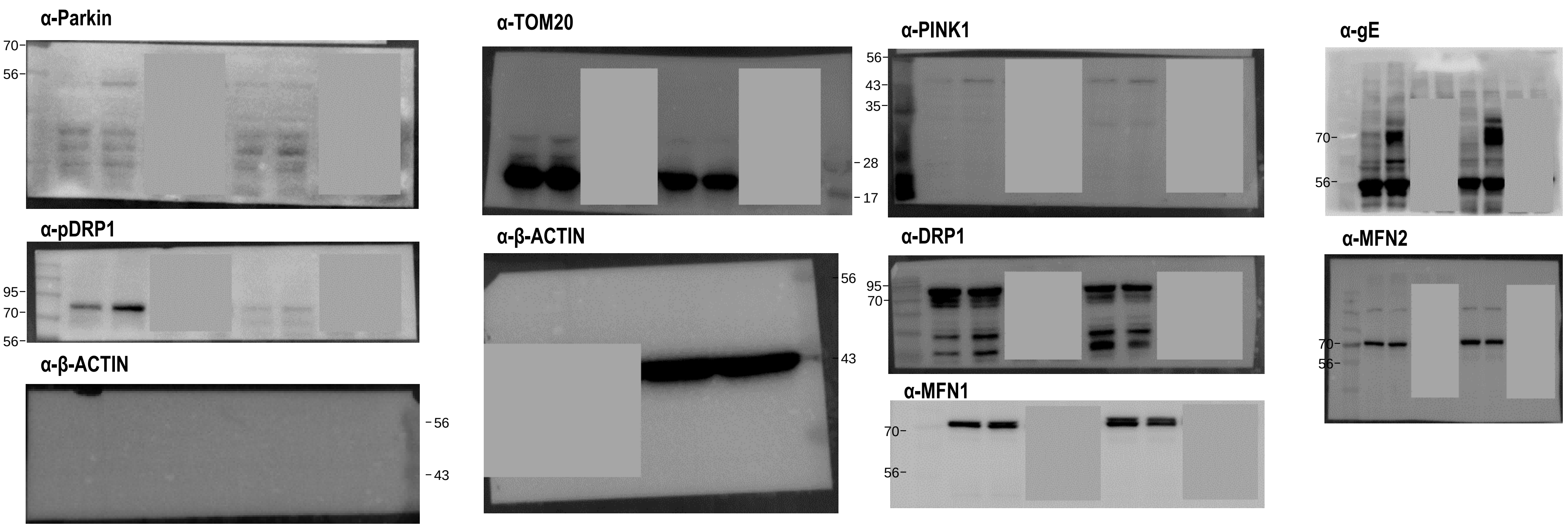
α-Parkin
70
56
α-TOM20
56
43
35
28
17
α-PINK1
α-gE
70
56
α-pDRP1
95
70
56
α-β-ACTIN
56
43
α-DRP1
95
70
α-MFN2
70
56
α-MFN1
70
56
α-β-ACTIN
56
43

FIG3C

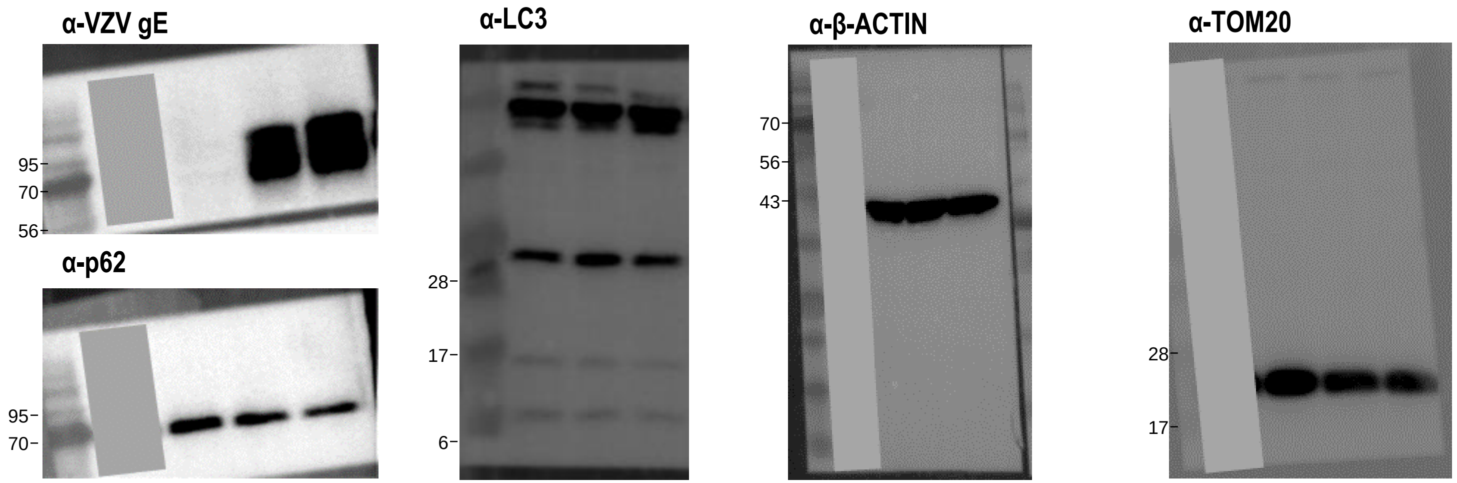
α-VZV gE
95
70
56
α-p62
95
70
α-LC3
28
17
6
α-β-ACTIN
70
56
43
α-TOM20
28
17

FIG3G

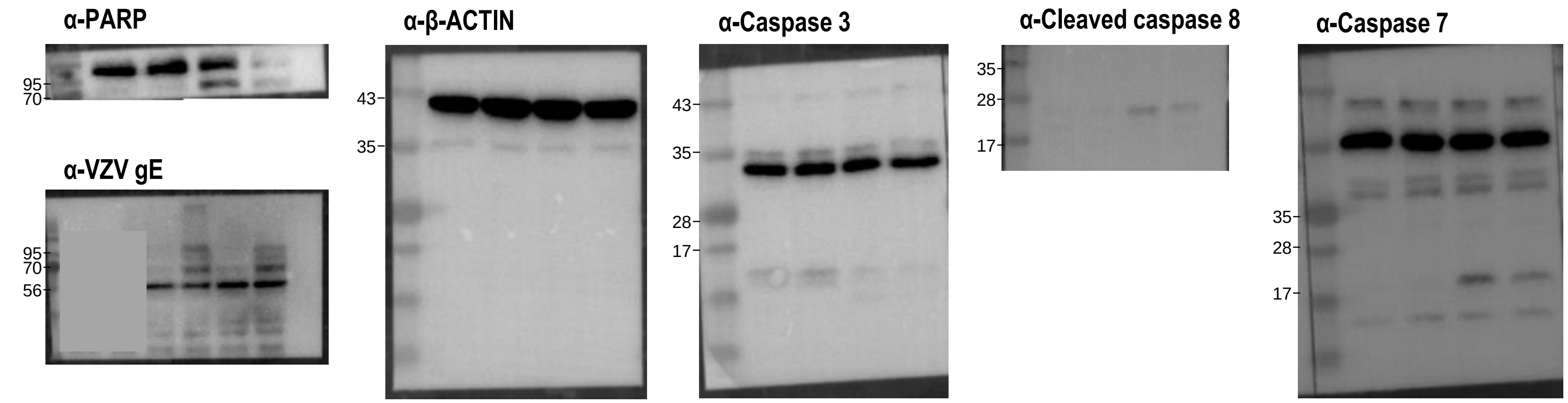

α-PARP
95
70
α-VZV gE
95
70
56
α-β-ACTIN
43
35
α-Caspase 3
43
35
28
17
α-Cleaved caspase 8
35
28
17
α-Caspase 7
35
28
17

FIG4A

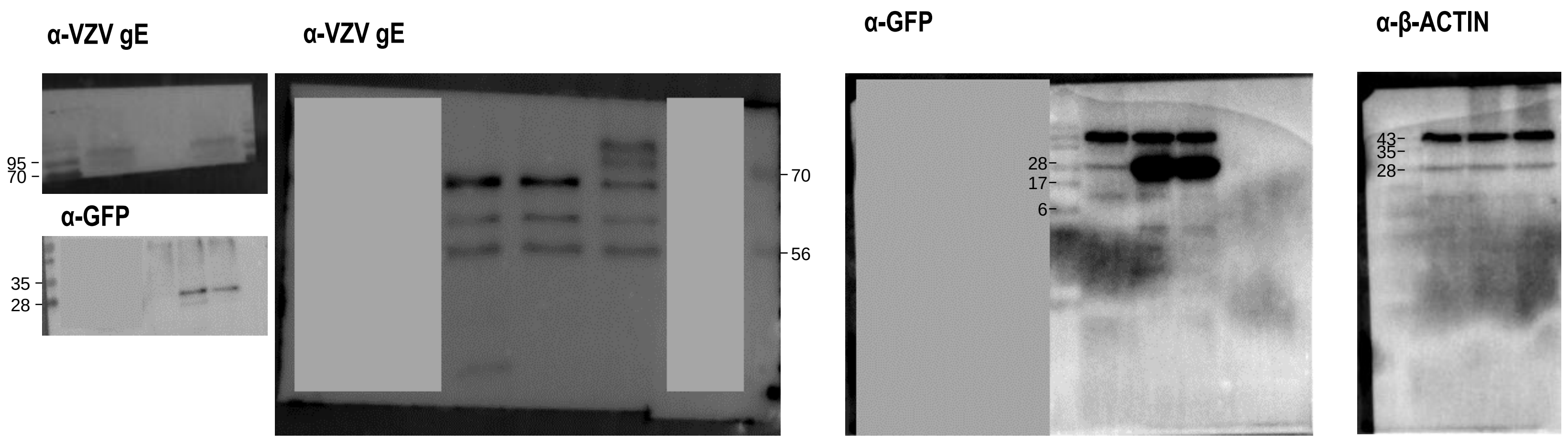

α-VZV gE
95
70
α-GFP
35
28
α-VZV gE
70
56
α-GFP
28
17
6
α-β-ACTIN
43
35
28

FIG4G

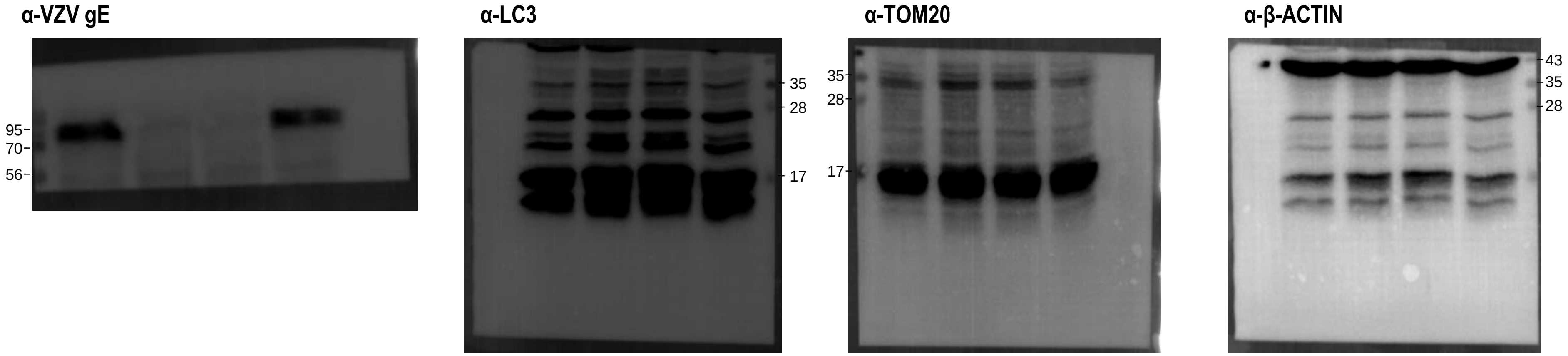
α-VZV gE
95
70
56
α-LC3
35
28
17
α-TOM20
35
28
17
α-β-ACTIN
43
35
28

FIG5B

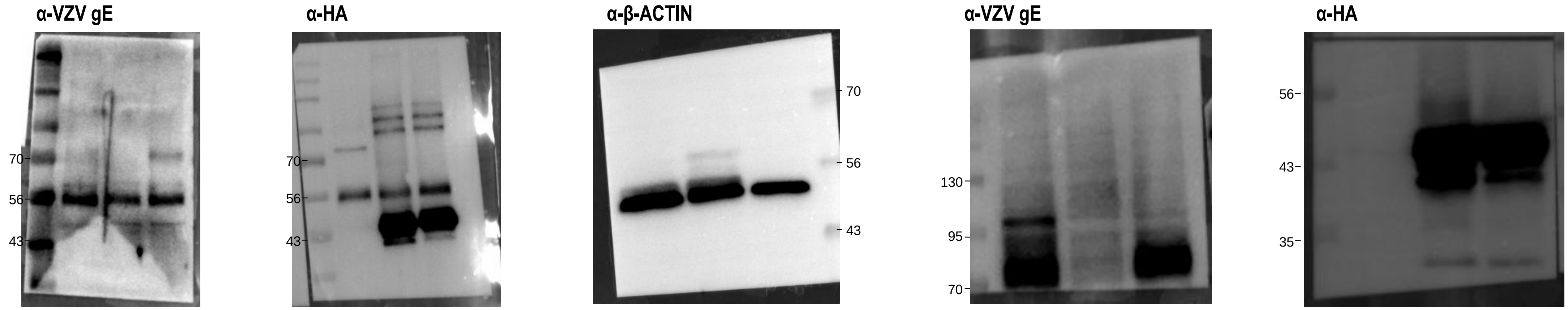
α-VZV gE
70
56
43
α-HA
70
56
43
α-β-ACTIN
70
56
43
α-VZV gE
130
95
70
α-HA
56
43
35

FIG5D

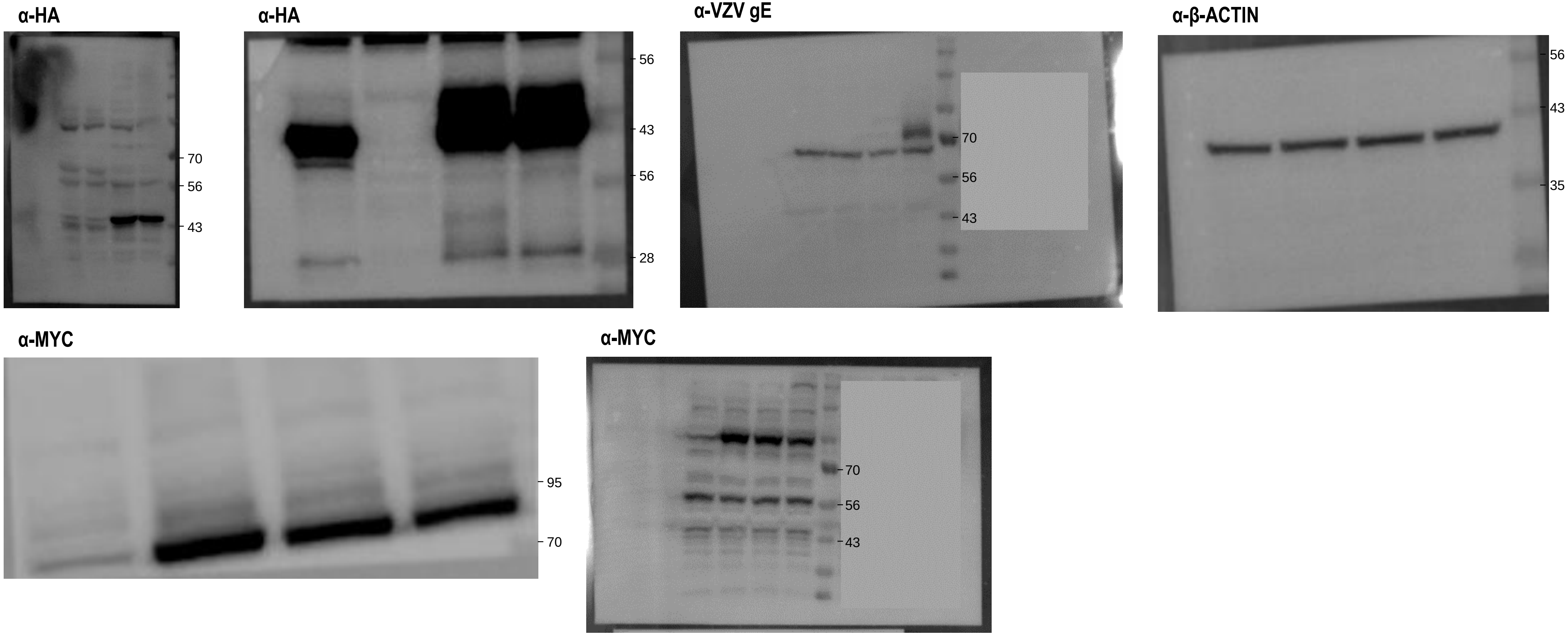
α-HA
70
56
43
α-HA
56
43
56
28
α-VZV gE
70
56
43
α-β-ACTIN
56
43
35
α-MYC
95
70
α-MYC
70
56
43

FIG5F

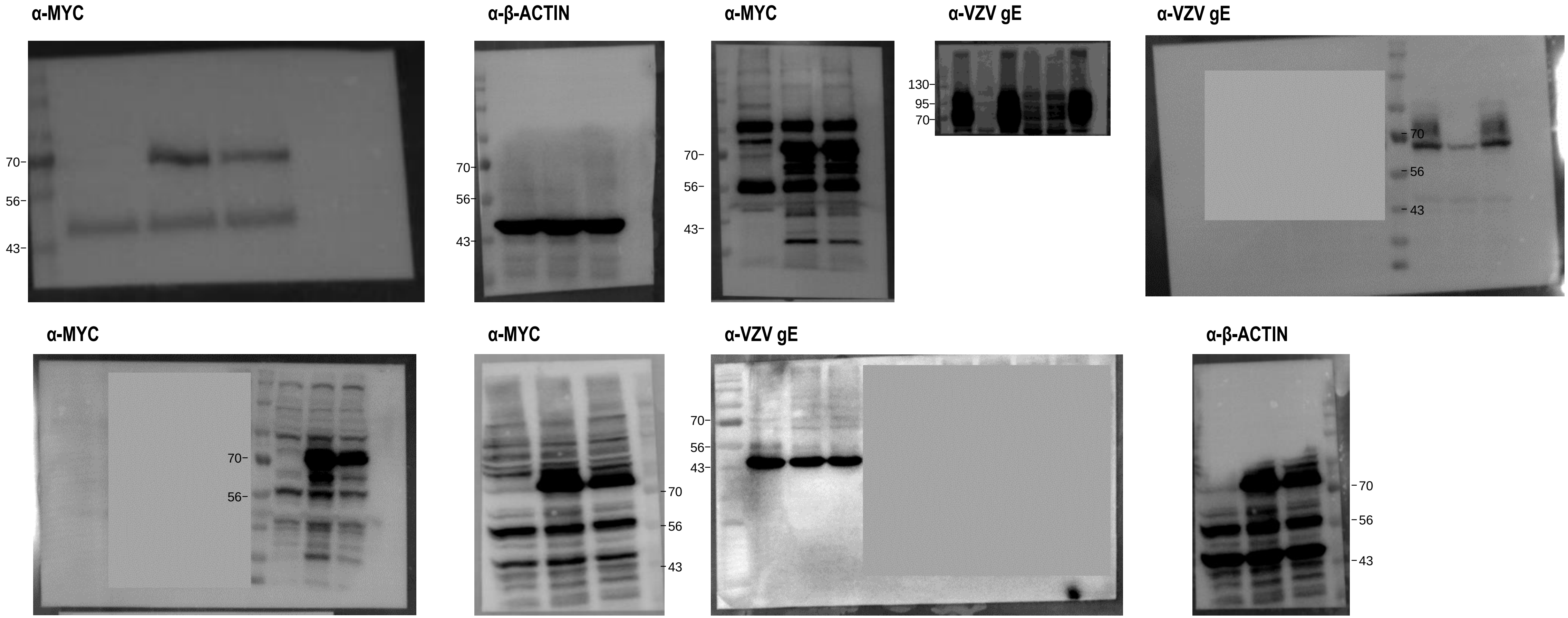
α-MYC
70
56
43
α-β-ACTIN
70
56
43
α-MYC
70
56
43
α-VZV gE
130
95
70
α-VZV gE
70
56
43
α-MYC
70
56
α-MYC
70
56
43
α-VZV gE
70
56
43
α-β-ACTIN
70
56
43

FIG5G

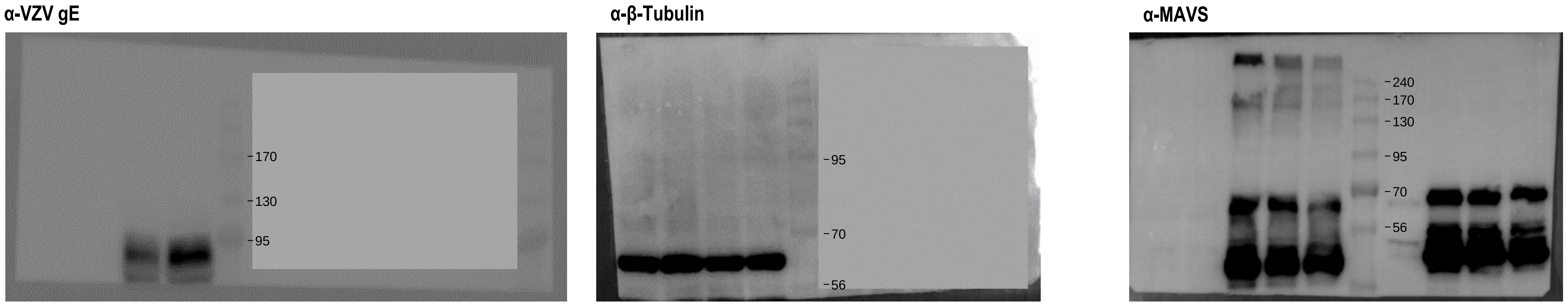
α-VZV gE
170
130
95
α-β-Tubulin
95
70
56
α-MAVS
240
170
130
95
70
56

FIG7D

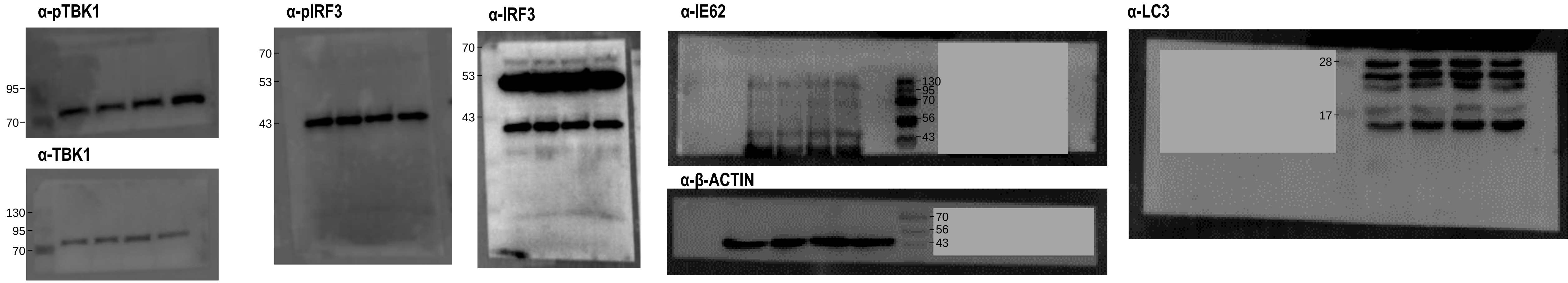
α-pTBK1
95
70
α-TBK1
130
95
70
α-pIRF3
70
53
43
α-IRF3
70
53
43
α-IE62
130
95
70
56
43
α-β-ACTIN
70
56
43
α-LC3
28
17
